# Immunogenetic Mechanisms Driving Norovirus GII.4 Antigenic Variation

**DOI:** 10.1371/journal.ppat.1002705

**Published:** 2012-05-17

**Authors:** Lisa C. Lindesmith, Martina Beltramello, Eric F. Donaldson, Davide Corti, Jesica Swanstrom, Kari Debbink, Antonio Lanzavecchia, Ralph S. Baric

**Affiliations:** 1 Department of Epidemiology, University of North Carolina, Chapel Hill, North Carolina, United States of America; 2 Institute for Research in Biomedicine, Bellinzona, Switzerland; 3 Humabs Biomed SA, Bellinzona, Switzerland; NIH, United States of America

## Abstract

Noroviruses are the principal cause of epidemic gastroenteritis worldwide with GII.4 strains accounting for 80% of infections. The major capsid protein of GII.4 strains is evolving rapidly, resulting in new epidemic strains with altered antigenic potentials. To test if antigenic drift may contribute to GII.4 persistence, human memory B cells were immortalized and the resulting human monoclonal antibodies (mAbs) characterized for reactivity to a panel of time-ordered GII.4 virus-like particles (VLPs). Reflecting the complex exposure history of the volunteer, human anti-GII.4 mAbs grouped into three VLP reactivity patterns; ancestral (1987–1997), contemporary (2004–2009), and broad (1987–2009). NVB 114 reacted exclusively to the earliest GII.4 VLPs by EIA and blockade. NVB 97 specifically bound and blocked only contemporary GII.4 VLPs, while NBV 111 and 43.9 exclusively reacted with and blocked variants of the GII.4.2006 Minerva strain. Three mAbs had broad GII.4 reactivity. Two, NVB 37.10 and 61.3, also detected other genogroup II VLPs by EIA but did not block any VLP interactions with carbohydrate ligands. NVB 71.4 cross-neutralized the panel of time-ordered GII.4 VLPs, as measured by VLP-carbohydrate blockade assays. Using mutant VLPs designed to alter predicted antigenic epitopes, two evolving, GII.4-specific, blockade epitopes were mapped. Amino acids 294–298 and 368–372 were required for binding NVB 114, 111 and 43.9 mAbs. Amino acids 393–395 were essential for binding NVB 97, supporting earlier correlations between antibody blockade escape and carbohydrate binding variation. These data inform VLP vaccine design, provide a strategy for expanding the cross-blockade potential of chimeric VLP vaccines, and identify an antibody with broadly neutralizing therapeutic potential for the treatment of human disease. Moreover, these data support the hypothesis that GII.4 norovirus evolution is heavily influenced by antigenic variation of neutralizing epitopes and consequently, antibody-driven receptor switching; thus, protective herd immunity is a driving force in norovirus molecular evolution.

## Author Summary

Noroviruses are the principal cause of epidemic gastroenteritis worldwide with GII.4 strains accounting for 80% of infections. The major capsid protein of GII.4 strains is evolving rapidly, resulting in new epidemic strains with altered antigenic sites. To define these sites we prepared the first human monoclonal antibodies (Hu mAbs) against GII.4 noroviruses by immortalizing memory B cells and characterizing antibody reactivity and carbohydrate blockade responses across a ∼20 year panel of time-ordered GII.4 virus-like particles (VLPs). Reflecting the complex exposure history of the patient, human anti-GII.4 mAbs grouped into three VLP reactivity patterns: broad (1987–2009), contemporary (2004–2009), and ancestral (1987–2002). We also identified the location of several defined epitopes which evolve over time and drive antigenic change. Our data indicate that antibodies targeting these sites block carbohydrate binding and likely select for the emergence of new strains that escape herd immunity and recognize unique carbohydrates for entry, resulting in new outbreaks of disease in vulnerable human populations. Importantly, these studies critically inform the rational design of broadly active vaccines and immunotherapeutics for the treatment of norovirus disease.

## Introduction

Noroviruses (NoVs) are the leading cause of severe viral gastroenteritis and are responsible for 50% of all acute gastroenteritis outbreaks in the United States and Europe [1]. Although the severity of disease is usually moderate, lasting 1–3 days, infection can be especially virulent in young children, the elderly, and the immunocompromised, with the latter group experiencing chronic diarrhea and virus shedding for over a year [2]–[8]. Importantly, it is estimated that 200,000 people die each year from norovirus infections, primarily children in the developing world [9]. An effective vaccine would be particularly advantageous for the very young and aged populations, military personnel, children and healthcare providers, food handlers, cruise ship passengers, and populations of the developing world [10]. Immunotherapeutics are especially needed for treating immunosuppressed populations experiencing long-term infections with chronic diarrhea. The lack of understanding of the extensive antigenic relationships among the large number of norovirus strains and the complex relationship between host protective immunity and virus antigenic heterogeneity are the primary obstacles to norovirus vaccine development.

Noroviruses are ∼38 nm icosahedral viruses with a ∼7.5 kb single-stranded, positive-sense RNA genome that contains three large open reading frames (ORFs). ORF1 encodes the non-structural proteins, while ORFs 2 and 3 encode the major and minor capsid proteins respectively. Expression of the major capsid protein (ORF2) in Venezuelan equine encephalitis (VEE) virus or baculovirus results in the formation of virus-like particles (VLPs) composed of 90 copies of the major capsid protein dimer [11]. Noroviruses are grouped by the amino acid sequence of the major capsid protein: viruses with less than 14.3% difference are classified as the same strain, 14.3–43.8% difference as the same genotype, and 45–61.4% difference as the same genogroup [12]. Currently, noroviruses are grouped into five genogroups (GI–GV). Genogroups GI and GII are responsible for most human infections and are further subdivided into 8 and 21 different genotypes, respectively [1], [12].

Structurally, the capsid monomer is divided into three domains. The shell domain (S) forms the core of the particle and the protruding domain (P) extends away from the core. The P domain is further subdivided into the P1 subdomain (residues 226–278 and 406–520) and the P2 subdomain (residues 279–405) [11]. The P2 subdomain is the most exposed region of the viral particle and is well positioned to interact with potential neutralizing antibodies and histoblood group antigen (HBGA) ligands [13]–[17]. Previous studies have shown that the P2 subdomain of the major capsid protein of GII.4 strains is evolving rapidly, resulting in new epidemic strains with altered carbohydrate ligand binding properties and antigenicity [13], [18]–[23].

For the past two decades, the majority of norovirus outbreaks have been caused by strains within the genogroup II, genotype 4 (GII.4 strains) subcluster. Between 1995 and 2006, four major norovirus pandemics associated with GII.4 strains were characterized using molecular epidemiologic methods. During the mid-1990's [24] strain US95/96 was responsible for ∼55% of the norovirus outbreaks in the USA and 85% of the outbreaks in the Netherlands [25]. In 2002, the US95/96 strain was replaced by the Farmington Hills strain [26], which was associated with ∼80% of norovirus outbreaks [27] in the USA. In 2004, the Hunter GII.4 variant was detected in Australia, Europe, and Asia [28]–[30]. Hunter strains were largely replaced in 2006 by two new co-circulating GII.4 variants in the USA and Europe, Laurens (2006a) and Minerva (2006b) [5], [29], [31]. Although similar to Minerva, Apeldoorn317 (GII.4.2008, GenBank accession no. AB445395) represents a new evolutionary cluster in the phylogeny of the GII.4 viruses. Most recently, a new GII.4.2006 variant, GII.4.2009 New Orleans, has been the predominate outbreak strain, although GII.4.2006 Minerva continues to circulate at low levels [1], [32].

A variety of studies using time ordered human outbreak sera and mouse monoclonal antibodies support the hypothesis that the GII.4 noroviruses are undergoing antigenic variation and that this variation contributes to the emergence of new outbreak strains over time [13], [22], [33], [34]. However, the lack of a cell culture or small animal model for human norovirus cultivation restricts study of neutralization antibodies and epitopes. To circumvent this problem, highly informative *in vitro* assays have been developed that measure the ability of an antibody to “block” binding of a VLP to a carbohydrate ligand [13], [35], [36]. This assay is highly sensitive, as it differentiates between norovirus strains too similar to be distinguished by enzyme immunoassay (EIA). The clinical relevance of the blockade assay, as a surrogate neutralization assay, has been confirmed in both infected chimpanzees [37] and Norwalk virus-infected humans [10], [38]. For NoV strains that hemagglutinate RBCs, high blockade antibody titers that prevent hemagglutination also correlate with protection from disease in humans [39]. Using human norovirus outbreak sera, VLP-immunized mouse sera, and mouse mAbs [13], [33], [34], the early GII.4 strains (1987 and 1997) were antigenically indistinguishable from each other by EIA and surrogate neutralization assays. VLPs of strains circulating post 2002 had significantly less reactivity with sera directed against earlier strains and no reactivity to mouse mAbs directed to GII.4.1987. Conversely, select mouse mAbs generated against GII.4.2006 reacted with VLPs that circulated only from 2002 or later. No blockade epitopes were found to be in common between GII.4.1987 and GII.4.2006.

Prior to this work, we and others had predicted GII.4 antibody epitopes using bioinformatic techniques. As expected, discreet amino acids are repeatedly identified as potential evolving epitopes. In particular, residues 296–298 and 393–395 are consistently identified as putative epitopes that change between epidemic GII.4 strains. Additional surface residues at 333, 340, 356, 368, 372, 407 and 412–413 were also predicted as potential antibody epitopes [13], [18], [19], [22], [28], [40]–[42]. These amino acids tend to cluster on loops and ridges of the P2 subdomain where antibody interaction would be most accessible. Beyond bioinformatics predictions, only a few studies have shown empirical evidence mapping GII.4 antigenic change. Allen *et al.* [42] compared the reactivity of five monoclonal antibodies against a pre and post-2002 epidemic GII.4 strain, and identified conformational epitopes composed of residues 294–296 and 393–395. The carbohydrate blockade potential of these antibodies was not reported, so the role of these sites in escape from herd immunity was unclear. However, the finding that residues 393–395 were antigenically important supported our previously published work identifying amino acid 395 as an antigenic determinant in the GII.4.2002 Farmington Hills strain [13]. Using mouse mAbs and molecular biology approaches to exchange predicted epitopes between GII.4 strain backbones, we have clearly identified amino acids 294, 296–298, 368 and 372 to comprise an evolving blockade epitope, as exchange of these amino acids from GII.4.1987 into GII.4.2006 conferred binding of mAbs that recognize GII.4.1987 but not GII.2006 [43]. Extending this approach, we have also confirmed amino acids 407, 412 and 413 to constitute a GII.4.2002 Farmington Hills-specific blockade epitope [17]. These empirical studies support the validity of using computational analysis to guide norovirus epitope studies.

Comparing reactivity of polyclonal sera collected from immunized mice and infected humans suggested antigenic variation within the GII.4 noroviruses [13], [33]. The development of mouse mAbs to different time-ordered GII.4 VLPs has greatly facilitated progress towards understanding the complex antigenic relations among these strains by clearly demonstrating antigenic variation over time and epidemic strain [34], [42], [43]. However, to maximally define the mechanistic relationships that exist between antigenic variation, immunity, and HBGA binding patterns noted in the GII.4 noroviruses in the context of natural infection history, the cross reactivity patterns, blockade responses, and epitope targets of human anti-GII.4 monoclonal antibodies are needed. Robust approaches exist for the isolation of human monoclonal antibodies that are elicited following virus infection. Using human PBMCs as a source of memory B cells, we created a panel of human mAbs directed against GII.4 strains and compared the reactivity of these mAbs to a panel of time-ordered GII.4 VLPs using EIAs and surrogate neutralization assays. We identified one novel, broadly cross reactive antibody that differentially blocks GII.4.1987 through 2009 VLP interactions with carbohydrate ligands, a potential immunotherapeutic for the treatment of acute or chronic GII.4 disease. We also defined unique antibody interactions with two different surface exposed epitopes that evolve over time. Importantly, antigenic variation in one of these epitopes correlated with changing carbohydrate ligand binding patterns over time, supporting the proposed relationship between epitope escape from human herd immunity and changing HBGA usage for virus docking [13]. In addition to defining the first human monoclonal antibodies with therapeutic potential for treating acute and chronic NoV GII.4 infections, these data support the hypothesis that GII.4 norovirus evolution results in antigenic drift of neutralizing epitopes and consequently, antibody-driven HBGA receptor switching; thus, protective herd immunity is a driving force in norovirus evolution.

## Results

### Donor sample characterization and mAb isolation

**Figure 1:**
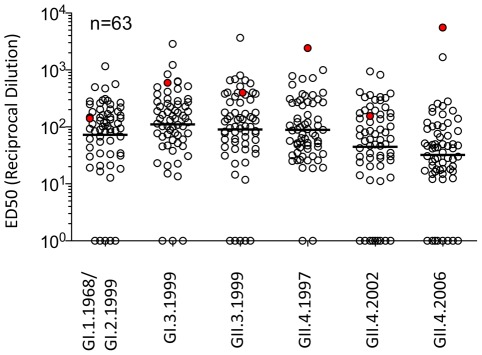
EIA Reactivity of plasma collected from healthy donors against norovirus VLPs. VLP-specific IgG titers in 63 plasma samples collected in early 2009 were measured by EIA using a panel of norovirus VLPs as antigen. Reciprocal ED50 dilutions (see Materials and Methods) are shown. Highlighted in red is the plasma sample of the donor NVB selected for the isolation of human mAbs. ED50 values below 10 were scored as negative and assigned a value of 1. The line shows the geometric mean value.

**Figure 2:**
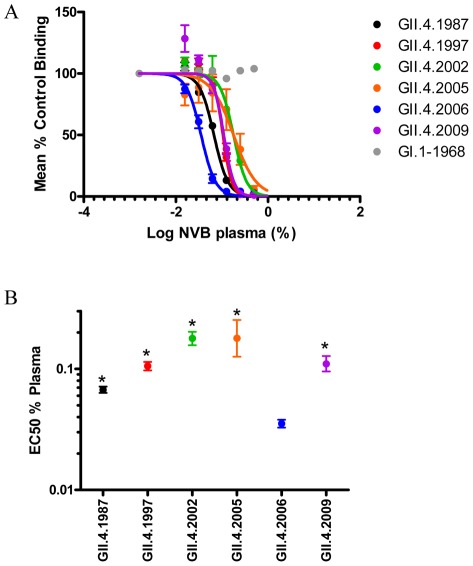
Characterization of donor NVB plasma blockade of norovirus VLPs. **Panel A**: PGM binding blockade activity. Sigmoidal curves were fit to the mean percent control binding calculated by comparing the amount of VLP bound to PGM in the presence of antibody pretreatment to the amount of VLP bound in the absence of antibody pretreatment. Error bars represent the standard error of the mean. **Panel B**: Mean EC50 (% plasma) for blockade of each VLP. * VLPs with EC50 values significantly different from the EC50 for GII.4.2006.

The development of mouse mAbs against different time-ordered GII.4 VLPs has greatly facilitated understanding of the complex antigenic relations between these strains by clearly demonstrating antigenic variation over time and by epidemic strain [34]. However, understanding of the human anti-GII.4 norovirus antibody response is essential not only for understanding the complex relationships between host immunity and virus antigenic change, but also for rational vaccine design based on defined neutralizing epitopes. Therefore, we developed a panel of human anti-GII.4 norovirus monoclonal antibodies to begin to characterize GII.4 antibody reactivity in the native virus host under natural infection conditions, noting that the norovirus pre-exposure histories in human volunteers are unknown and can only be inferred by human sera cross-reactive antibody binding and blockade patterns using time-ordered VLPs representing different outbreak and pandemic strains (Table 1) [13], [33]. Plasma and PBMC samples from 63 healthy individuals were collected in early 2009 and plasma binding titers (ED50) were measured by EIA against a panel of 6 different norovirus VLPs representing GI and GII strains (Figure 1). The majority of tested samples reacted by EIA with variable ED50 titers to the panel of VLPs tested (Figure S1). One sample (Donor NVB) was shown to react strongly with GII VLPs and was therefore chosen for further characterization and isolation of norovirus-specific mAbs. The NVB plasma sample was tested again by EIA against a larger panel of norovirus VLPs and shown to react with the entire panel of GII.4 VLPs representing epidemic strains from 1987 to 2009 (Table 2 and Figure S2). Plasma did not react with GI.1 VLPs. Plasma reactivity to VLPs representing genogroup II strains outside the GII.4 genocluster was variable by strain. NVB plasma efficiently blocked pig gastric mucin (PGM) binding of each of the GII.4 VLPs (Figure 2). However, significantly less plasma was needed to block GII.4.2006 binding to PGM (EC50 0.0353% plasma) than was needed to block the other GII.4 VLPs binding to PGM (EC50 range 0.0673–0.1791% plasma), supporting an association between the donor plasma titers and the prevalence of global circulating NoV strains. Having demonstrated antibody responses to the complete GII.4 panel, memory B cells from this PBMC donor were EBV-immortalized and seven anti-GII.4 mAbs were isolated and characterized. Of note, all seven mAbs were isotype IgG1, agreeing with previous observations of a predominantly T_h1_ mediated immune response to norovirus in challenged volunteers (data not shown and [36], [44]). None of the antibodies recognized GI VLPs (data not shown and Table 2 and Figure S2). EIA reactivity was limited to GII.4 strains for mAbs NVB 114, 97, 111, 43.9 and 71.4. Monoclonal Abs 37.10 and 61.3 extended reactivity to include additional VLPs from GII.1, GII.2 and GII.12 genoclusters (Table 2 and Figure S2). The reactivity of mAbs between GII.4 VLPs varied, but could be grouped into time-related clusters for four of the seven human mAbs. The remaining three mAbs demonstrated broad GII.4 reactivity.

**Table 1:** NoV Strains (VLPs) used in this study.

VLP	Pandemic Cluster	GenBank Access. No.	Original Strain
**GI.1.1968**	None	M87661	Outbreak Isolate
**GI.1.2001**	None	AY502016	Hu/NoV/West Chester/2001/USA
**GI.2.1999**	None	JQ743332	Outbreak Isolate
**GI.3.1999**	None	JQ743330	Outbreak Isolate
**GI.4.2000**	None	JQ743331	Outbreak Isolate
**GII.1.1971**	None	U07611	Outbreak Isolate
**GII.2.1976**	None	AY134748	Outbreak Isolate
**GII.2.2002**	None	AB195225	Hu/GII/Ina/02/JP
**GII.3.1999**	None	JQ743333	Outbreak Isolate
**GII.4.1987**	Ancestral	AAK50355.1	Hu/NLV/GII/MD14512/1987/US
**GII.4.1997**	US95/96	JQ478407	Outbreak Isolate
**GII.4.2002**	Farmington Hills	JQ478408	Outbreak Isolate
**GII.4.2004**	Hunter	AAZ31376.2	Hu/GII.4/Hunter284E/04O/AU
**GII.4.2005**	None	BAE98194.1	Hu/Sakai/04/179/2005/JP
**GII.4.2006**	Minerva/2006b	JQ478409	Outbreak Isolate
**GII.4.2009**	Minerva/2006b	ADD10375	Hu/GII.4/New_Orleans1805/2009/USA
**GII.12.2010**	None	HQ401025	Hu/GII.12/HS207/2010/USA

**Table 2:** NVB plasma and monoclonal antibody EIA reactivity to norovirus VLPs.

NVB Antibody	Plasma	114	97	111	43.9	37.10	61.3	71.4
**VLP**								
**GI.1.1968**	−	−	−	−	−	−	−	−
**GI.1.2001**	−	−	−	−	−	−	−	−
**GII.1.1971**	+	−	−	−	−	+	+	−
**GII.2.1976**	−	−	−	−	−	+	+	−
**GII.2.2002**	−	−	−	−	−	+	+	−
**GII.3.1999**	−	−	−	−	−	+	+	−
**GII.12.2010**	+	−	−	−	−	+	+	−
**GII.4.1987**	+	+	−	−	−	+	+	+
**GII.4.1997**	+	+	−	−	−	+	+	+
**GII.4.2002**	+	−	−	−	−	+	+	+
**GII.4.2004**	+	−	+	−	−	+	+	+
**GII.4.2005**	+	−	+	−	−	+	+	+
**GII.4.2006**	+	−	+	+	+	+	+	+
**GII.4.2009**	+	−	+	−	+	+	+	+

+; mean optical density after background subtraction for VLP-coated wells was greater than three times the mean optical density for PBS-coated wells.

### Characterization of a human mAb specific for early GII.4 norovirus strains

**Figure 3:**
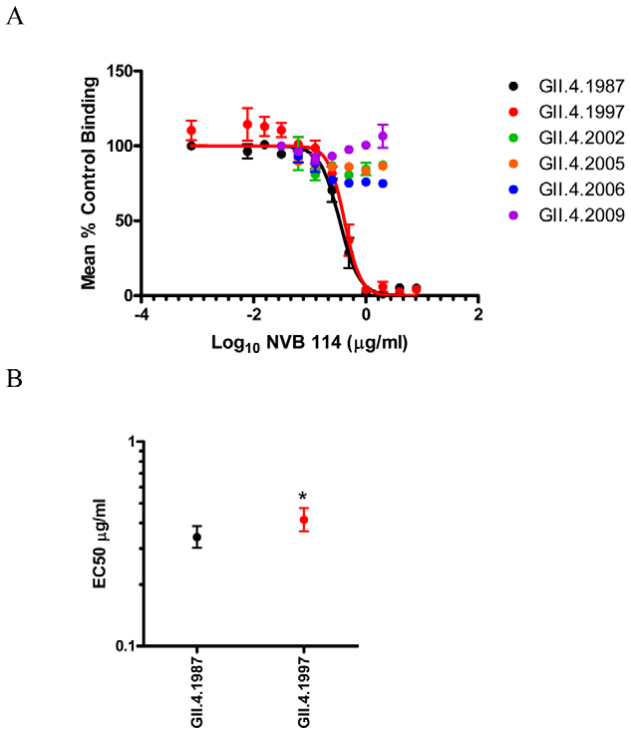
Human mAb NVB 114 recognizes a blockade epitope restricted to early GII.4 strains. **Panel A**: PGM binding blockade activity. Sigmoidal curves were fit to the mean percent control binding calculated by comparing the amount of VLP bound to PGM in the presence of antibody pretreatment to the amount of VLP bound in the absence of antibody pretreatment. Error bars represent the standard error of the mean. **Panel B**: Mean EC50 (µg/ml) for blockade of each VLP. * VLP with EC50 value significantly different from the EC50 for GII.4.1987.

Human mAb NVB 114 reacted by EIA and blockade assay exclusively with GII.4.1987 and GII.4.1997 (Table 2, Figure 3, and Figure S2). Significantly more antibody was needed to block GII.4.1997-PGM binding (EC50 0.4152 µg/ml) than GII.4.1987-PGM binding (EC50 0.3414 µg/ml) (Figure 3B) (p<0.05), supporting the hypothesis that subtle antigenic differences exist between these strains.

### Characterization of a human mAb specific for contemporary GII.4 norovirus strains

**Figure 4 ppat-1002705-g004:**
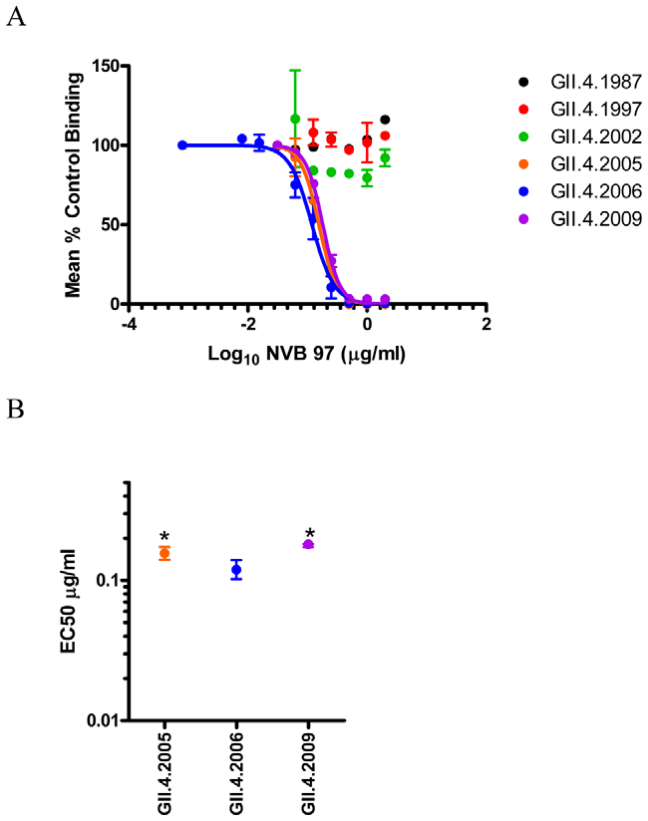
Human mAb NVB 97 recognizes a blockade epitope restricted to contemporary GII.4 strains. **Panel A**: PGM binding blockade activity. Sigmoidal curves were fit to the mean percent control binding calculated by comparing the amount of VLP bound to PGM in the presence of antibody pretreatment to the amount of VLP bound in the absence of antibody pretreatment. Error bars represent the standard error of the mean. **Panel B**: Mean EC50 (µg/ml) for blockade of each VLP. * VLP with EC50 value significantly different from the EC50 for GII.4.2006.

In contrast to the early strain GII.4 reactivity of NVB 114, EIA of human mAb NVB 97 exclusively recognized VLPs of contemporary circulating (2004–2009) GII.4 strains (Table 2 and Figure S2); VLPs representing GII.4 strains circulating prior to 2004 were not recognized by NVB 97. Accordingly, the NVB 97 blocked VLP-PGM interaction of GII.4.2005, 2006 and 2009 VLPs (Figure 4). A comparable blockade assay for GII.4.2004 is not available, as our strain doesn't bind carbohydrate ligand under our conditions of treatment [13], [17], [34]. However, under standard conditions, the EC50 for GII.4.2006 (0.1195 µg/ml) was significantly less than the EC50 of GII.4.2005 (0.1559 µg/ml) and GII.4.2009 (0.1810 µg/ml) (Figure 4B) (p<0.05). These data are consistent with the hypothesis that the contemporary 2009 Minerva variant may be diverging antigenically from its 2006 Minerva variant ancestral strain.

### Characterization of human mAbs specific for Minerva variant strains

**Figure 5 ppat-1002705-g005:**
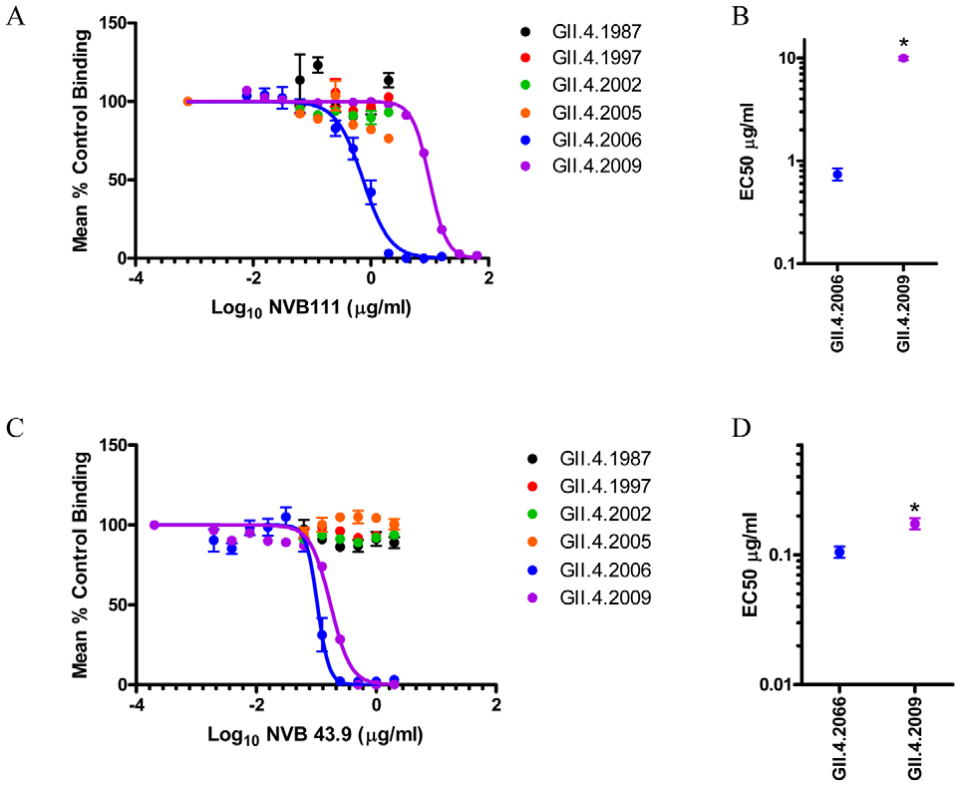
Human mAbs NVB 111 and 43.9 recognize a blockade epitope restricted to Minerva variants. **Panel A (NVB 111) and C (NVB 43.9)**: PGM binding blockade activity. Sigmoidal curves were fit to the mean percent control binding calculated by comparing the amount of VLP bound to PGM in the presence of antibody pretreatment to the amount of VLP bound in the absence of antibody pretreatment. Error bars represent the standard error of the mean. **Panels B (NVB 111) and D (NVB 43.9)**: Mean EC50 (µg/ml) for blockade of each VLP. * VLP with EC50 value significantly different from the EC50 for GII.4.2006.

The difference in blockade sensitivity of GII.4.2006 and GII.4.2009 to NVB 97 provides the first evidence of subtle antigenic divergence between two Minerva variants, each of which caused widespread outbreaks globally [1]. This observation is further supported by Human mAbs NVB 111 and NVB 43.9 reactivity profiles. By single-dilution EIA, NVB 111 specifically reacted to 2006 but minimally with the 2009 variant of Minerva and other tested VLPs (Table 2 and Figure S2). Accordingly, NVB 111 required 13-fold more antibody to block GII.4.2009-PGM interaction (EC50 9.953 µg/ml) than it required to block GII.4.2006-PGM interaction (EC50 0.7376 µg/ml) (Figure 5A and B) (p<0.05). In comparison, NVB 43.9 specifically recognized both the 2006 and 2009 Minerva variants by EIA (Table 2 and Figure S2). The interaction of both variants with PGM ligand was efficiently blocked by NVB 43.9 (Figure 5C and D). At relatively low antibody concentrations, NVB 43.9 did significantly differentiate GII.4.2006 from GII.4.2009 (EC50 0.1031 and 0.1739 µg/ml, respectively). Combined, these three human mAbs (NVB 97, 111, and 43.9) indicate that GII.4.2006 and GII.4.2009 are diverging from each other at the antigenic level, but that significant 2006 blockade epitopes are still preserved, suggesting that additional evolution is needed prior to the emergence of an antigenically distinct, new pandemic strain.

### Characterization of broadly-reactive human anti-GII.4 mAbs

**Figure 6:**
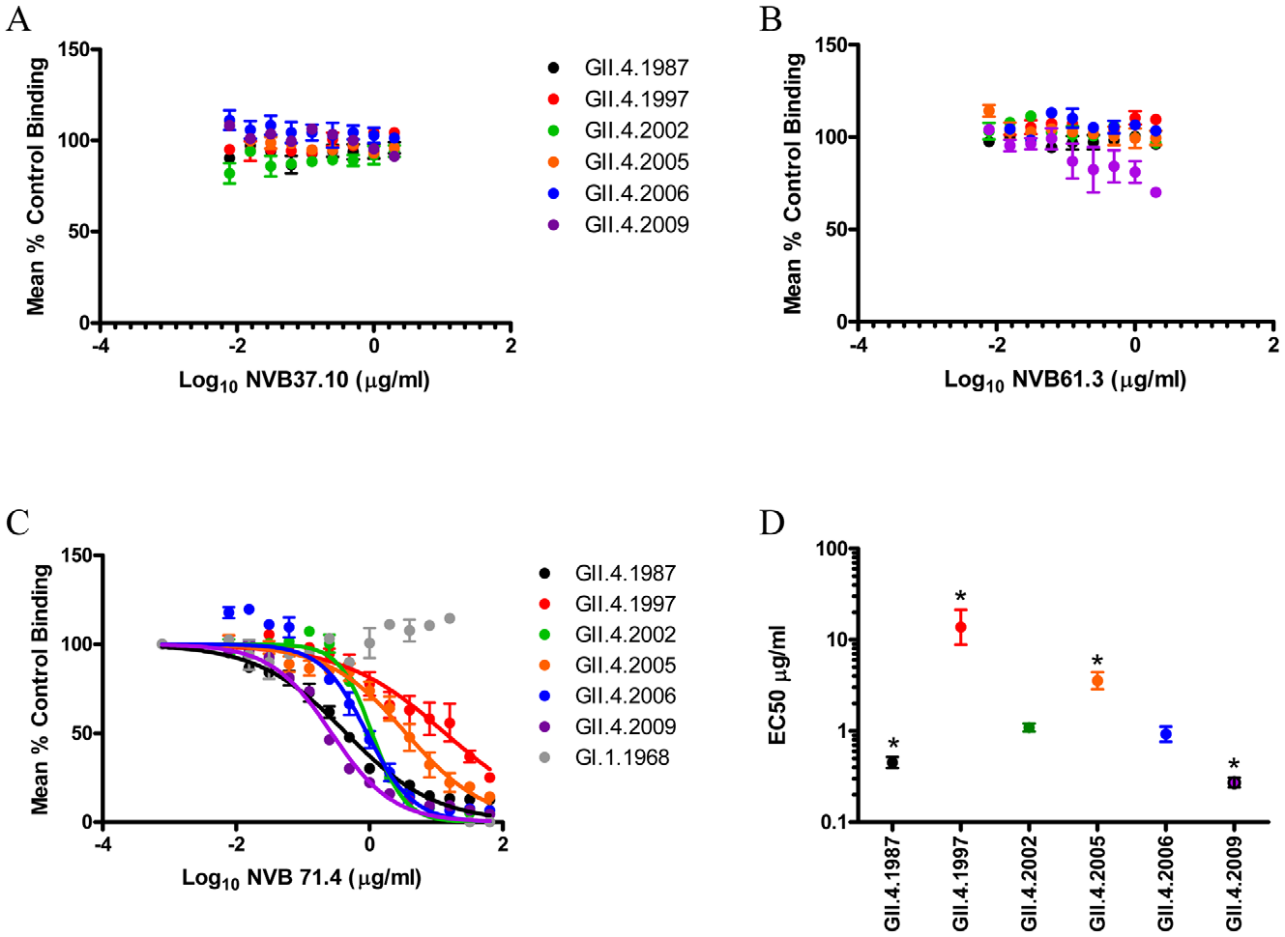
Human mAbs NVB 37.10, 61.3 and 71.4 recognize a conserved epitope. PGM binding blockade activity of NVB 37.10 (**Panel A**), NVB 61.3 (**Panel B**) and NVB 71.4 (**Panel C**). Sigmoidal curves were fit to the mean percent control binding calculated by comparing the amount of VLP bound to PGM in the presence of antibody pretreatment to the amount of VLP bound in the absence of antibody pretreatment. Error bars represent the standard error of the mean. **Panel D**: Mean EC50 (µg/ml) for blockade of each VLP by NVB 71.4. * VLPs with EC50 value significantly different from the EC50 for GII.4.2006.

NVB 114, 97, 111, and 43.9 recognize blockade epitopes that are evolving over time (Figures 3–5). Three additional antibodies recognize epitopes that are highly conserved over time. Human mAbs NVB 37.10 and 61.3 exhibited broad GII reactivity, detecting VLPs from GII.1, GII.2, GII.3 and GII.12 genoclusters and the entire panel of time-ordered GII.4 (1987–2009) VLPs (Table 2 and Figure S2). Despite broad EIA reactivity, NVB 37.10 and 61.3 did not efficiently block VLP-PGM interactions for any GII.4 VLP tested (Figures 6A and B). In contrast, human mAb NVB 71.4 recognized the entire time-ordered GII.4 VLP panel but was unreactive with any other GII VLPs (Table 2 and Figure S2). Remarkably, NVB 71.4 blocked VLP-PGM interaction of each of the GII.4 VLPs (Figure 6C). The blockade potential varied between the VLPs. GII.4.2002 and GII.4.2006 had similar EC50 values (1.095 and 0.9233 µg/ml, respectively), while significantly less antibody was needed for blockade of GII.4.1987 (EC50 0.4506 µg/ml) and GII.4.2009 (EC50 0.2716 µg/ml) and significantly more antibody was needed to block GII.4.1997 (EC50 13.73 µg/ml) and GII.4.2005 (EC50 3.544 µg/ml) (Figure 6D). These three human mAbs indicate the existence of conserved GII.4 epitopes over the past twenty-five years and across three pandemic strains that could serve as targets for broad-based vaccine design. Importantly, NVB 71.4 represents the first potential, broad spectrum immune-therapeutic for any NoV.

### Confirmation of mAb blockade phenotypes by alternative approaches

**Figure 7:**
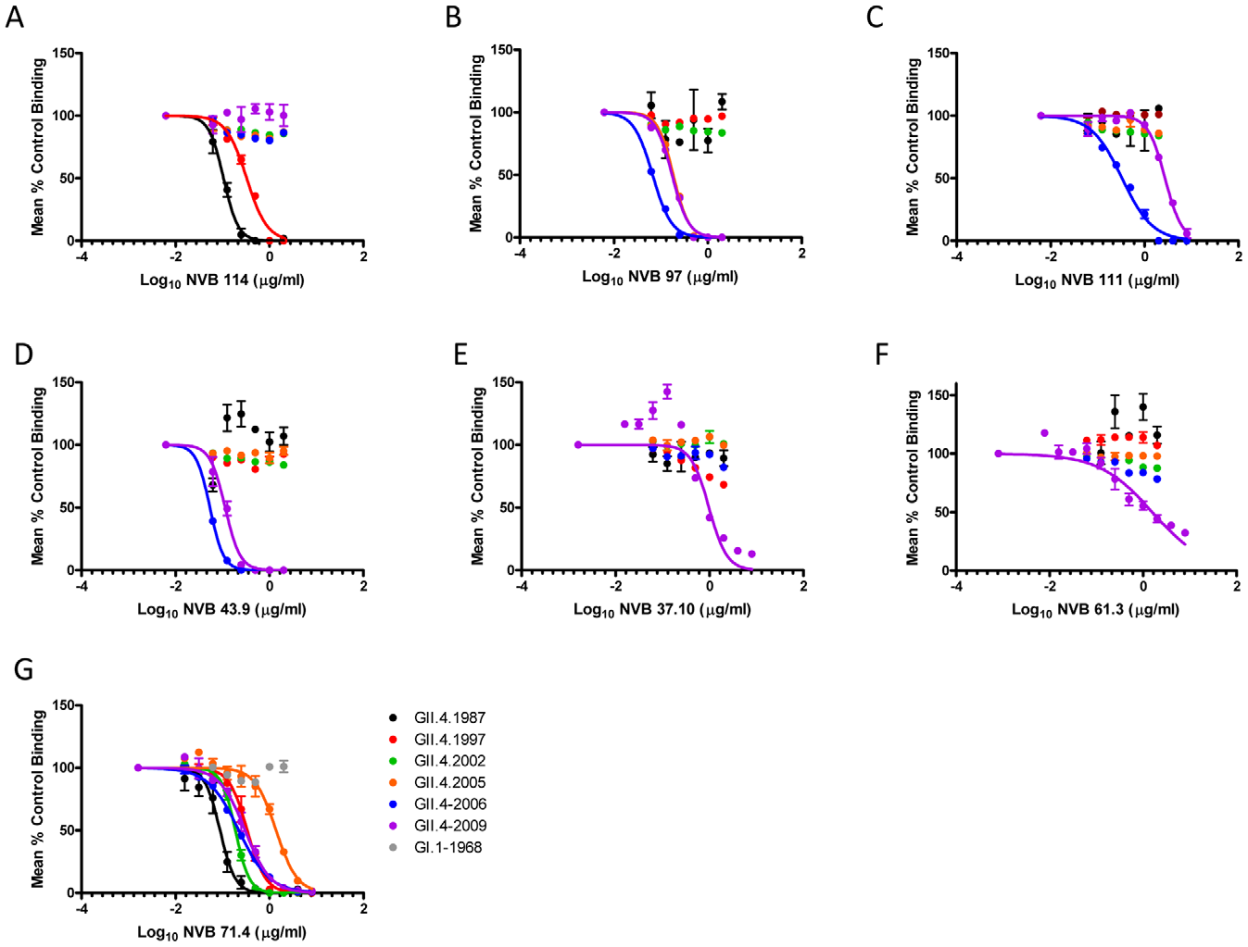
Blockade of Bi-HBGAs by Hu mAbs. Bi-HBGA binding blockade activity of NVB 114 (**Panel A**), NVB 97 (**Panel B**), NVB 111 (**Panel C**), NBV 43.9 (**Panel D**), NVB 37.10 (**Panel E**), NVB 61.3 (**Panel F**) and NVB 71.4 (**Panel G**). Sigmoidal curves were fit to the mean percent control binding calculated by comparing the amount of VLP bound to Bi-HBGA in the presence of antibody pretreatment to the amount of VLP bound in the absence of antibody pretreatment. Error bars represent the standard error of the mean. EC50 values are reported in Table S2.

In addition to VLP-PGM interaction blockade assay, human mAbs were also tested for blockade of VLP-synthetic biotinylated HBGA (Bi-HBGA) interaction and ability to block VLP hemagglutination of O+ RBCs. Regardless of substrate (PGM or Bi-HBGA), the dose-response profiles for all blockade antibodies and VLPs were similar (compare Figures 3–6 to Figure 7). Reflecting valency differences in the number of potential binding sites of HBGA in the two substrates, the EC50 values differed between assays (compare Tables S1 and S2). NVB 114 blocked only GII.4.1987 (EC50 0.1054 µg/ml) and GII.4.1997 (EC50 0.3275 µg/ml), with 1997 blockade requiring significantly more mAb (p<0.05). NVB 97 blocked only GII.4.2005 (EC50 0.1835 µg/ml), 2006 (EC50 0.0668 µg/ml), and 2009 (EC50 0.1732 µg/ml) with blockade of GII.4.2005 and 2009 requiring significantly more mAb than the blockade of GII.4.2006 (p<0.05). NVB 111 and 43.9 blocked only GII.4.2006 (EC50 0.3324 and 0.05406 µg/ml, respectively) and 2009 (EC50 2.727 and 0.1140 µg/ml, respectively) with significantly more mAb needed to block 2009 for both antibodies (p<0.05). Importantly, the broad blockade phenotype of NVB 71.4 was reproduced in the Bi-HBGA blockade assay. The EC50 value varied by VLP and ranged from 0.0906 µg/ml for GII.4.1987 to 1.219 µg/ml for GII.4.2005. Agreeing with the PGM assay, only GII.4.2002 (EC50 0.1679 µg/ml) and GII.4.2006 (EC50 0.2039 µg/ml) were blocked similarly (p>0.05). NVB 37.10 and 61.3, the two mAbs that did not block PGM interaction of any tested VLP, both blocked GII.4.2009 interaction with synthetic Bi-HBGAs by at least 50% (Figure 7E and F), although EC50 titers were relatively high (EC50 0.9753 and 1.581 µg/ml for NVB 37.10 and 61.3, respectively), compared to the amount of antibody needed to block GII.4.2009 by the strain-specific mAbs (EC50 0.1140 and 0.1732 µg/ml for NVB 43.9 and 97). Repeated testing with PGM as substrate did not replicate the findings with synthetic carbohydrate substrates (Figure 6A and B).

An additional measurement of antibody blockade ability uses RBCs as the VLP binding substrate. Previous work has demonstrated that Norwalk virus VLPs hemagglutinate (HA) O+ RBCs, that this interaction can be disrupted by antibodies found in polyclonal serum (hemagglutination inhibition; HAI), and that the HAI titer of serum correlates with antibody blockade of VLP-Bi-HBGA interaction [38], [39], [45]. To determine if these findings could be extended to study GII.4 VLP blockade, we first tested each GII.4 VLP for hemagglutination ability. In contrast to Norwalk VLPs, which demonstrated robust HA activity, VLPs of GII.4.1987, 1997, and 2009 did not reproducibly HA O+ RBCs (data not shown). GII.4.2002, 2005 and 2006 did HA O+ RBCs, and VLP HA was inhibited by NVB plasma (HAI 0.01% plasma for GII.4.2002 and 2005 and 0.001% plasma for GII.4.2006) (Table S3). Neither NVB plasma nor any of the mAbs inhibited Norwalk virus VLP HA. In agreement with the other two blockade assays, GII.4.2006 VLP HA of O+ RBCs was blocked by NVB 97 (HAI 0.07 µg/ml), 111 (HAI 0.5 µg/ml), and 43.9 (HAI 0.04 µg/ml) while HA of O+ RBCs by GII.4.2002 was unaffected by these mAbs at 0.5 µg/ml. NVB 97 also inhibited GII.4.2005 HA (HAI 0.13 µg/ml) but not GII.4.2002. The HAI profile of the cross-reactive mAbs had a weaker correlation with the blockade assays. NVB 71.4 and 37.10 each inhibited HA of GII.4.2005 (HAI 0.5 and 0.25 µg/ml) and 2006 (HAI 0.13 and 0.25 µg/ml). NBV 61.3 only inhibited HA of GII.4.2002 at 0.25 µg/ml.

### Macro scale evaluation of mAb epitopes

**Figure 8:**
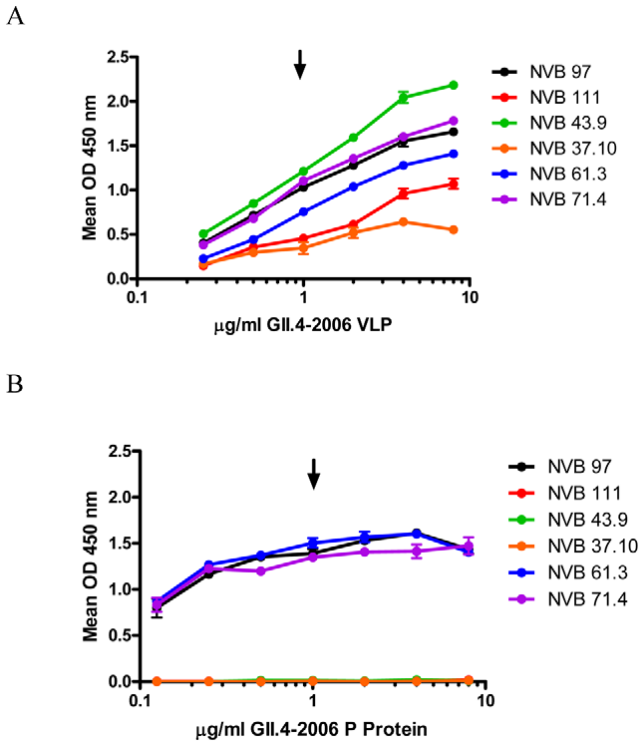
EIA Reactivity of mAbs to GII.4.2006 VLPs and P proteins. Increasing concentrations of GII.4.2006 VLP (**Panel A**) or P protein (**Panel B**) were assayed for reactivity to the hu mAbs by EIA. Arrow indicates 1 µg/ml, the concentration of GII.4.2006 VLP scored as postive by EIA (Figure S2 and Table 2). Bars are SEM.

Our previous work with mouse-derived anti-norovirus mAbs suggested that blockade epitopes are conformation dependent [17], [34]. To test the effect of protein conformation of human mAb binding, we used both Western blot and EIA analysis to compare antibody binding to GII.4.2006 VLPs and P proteins. P proteins of GII.4.2006 are composed of the C-terminal portion of the major capsid protein (amino acids 221–531) [21]. Expression of the P protein in *E. coli* results in small particle formation estimated to consist of 12 P dimers that reportedly maintains VLP characteristics in carbohydrate and antibody binding studies [46], [47]. None of the human anti-GII.4 mAbs recognized either the denatured VLP or P protein by Western blot analysis, suggesting that the epitopes for these antibodies are conformation dependent (data not shown). Surprisingly, only half of the mAbs that recognized GII.4.2006 VLP (Figure 8A) by EIA also recognized GII.4.2006 P protein by EIA (Figure 8B). NVB 71.4 and 61.3 extended their broad reactivity to P proteins, whereas NVB 37.10 did not, indicating that a minimum of three GII.4 cross-reactive epitopes must exist. NVB 97 also detected P protein by EIA. Neither of the Minerva variant mAbs recognized P protein even at protein concentrations 8-fold above standard EIA conditions (1 µg/ml coating protein). Further, all seven mAbs detected increasing concentrations of VLP in a linear dose response with signals saturating at 4 µg/ml of VLP when the mAb concentration was held at 1 µg/ml, which is our standard EIA antibody titer (Figure 8A). Antibody reactivity to the P protein saturated at a lower protein concentration than VLP and at optical densities below the linear range of the assay (compare Figure 8A and 8B), suggesting that even among the mAbs that bind to P proteins conformation-based epitopes may be limited in a way not observed with VLPs. These data suggest two important points. First, some of the mAb epitopes are highly sensitive to conformation, and secondly, that the principle P protein conformation is not identical to VLPs at least for some critical blockade epitopes.

### Predicting putative GII.4 evolving antibody epitopes

**Figure 9:**
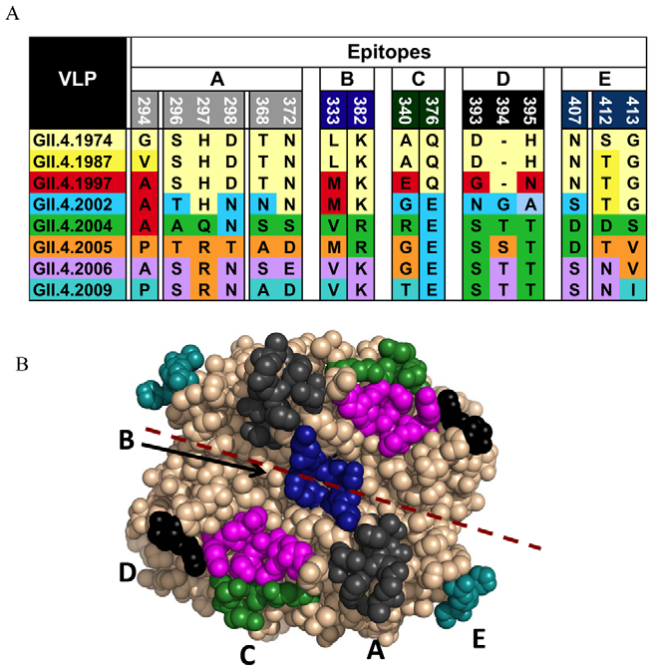
Predicted GII.4 norovirus evolving blockade epitopes. Bioinformatic approaches predicted five antibody epitopes on the surface of GII.4 noroviruses that appeared to be evolving over time and to correlate with the emergence of new GII.4 outbreak strains. **Panel A**: Amino acid variation of Epitopes A–E by GII.4 strain. **Panel B**: Predicted epitopes were expanded to include interacting amino acids within 8A. Epitope A (grey), Epitope B (blue), Epitope C (green), Epitope D (black), Epitope E (teal) and HBGA binding sites (magenta) mapped onto the P domain dimer of GII.4.2002.

The evolution of the GII.4 noroviruses was assessed over a 36-year period of time by comparing strains from 1974 to 2010. In comparing these sequences, sites of variation in the P2 subdomain were noted, and these sites were mapped onto the crystal structure of the P-domain dimer for the 1997 strain VA387. Surface-exposed sites of variation were then examined to determine which residues may be close enough to constitute a single epitope, and five epitopes were predicted based upon this variation (Figure 9A, and [40]).

Epitope A encodes significant amino acid changes over time and has also been demonstrated to be an evolving GII.4 blockade epitope using mouse mAbs (Figure 9A, 9B and [43]). Epitope A is conformational and is located on the top of the capsid proximal to the HBGA binding pocket. Six variable sites were close to each other in the region of this putative epitope, suggesting that these residues may work in concert to change the local structure of Epitope A. The variable, surface-exposed residues include positions 294, 296–298, 368 and 372. Of note, Epitope A is continuing to evolve in extant strains, whereby the amino acid at position 294 seems to vary extensively in strains from 2008–2010 (amino acid replacements P294A, P294S and P294T have been observed at this position). Epitope B was predicted based upon two variable residues at positions 333 and 382. While these residues are buried in the dimer interface between two chains, the patterns of variation at these sites suggest that they play an important role in the evolution of novel strains, perhaps by evolving replacements that allow the more surface exposed residues in other surface exposed epitopes to dramatically change the physiochemical properties of the amino acid replacements. Residues 340 and 376 make up the variable residues of putative Epitope C. This putative conformation dependent epitope is on the surface and lateral edge of the capsid and is directly proximal to the HBGA binding pocket, suggesting that this epitope may play a role in receptor switching along with Epitope D. Epitope D is comprised of three variable residues from positions 393–395. In the first reported crystal structure for the GII.4 noroviruses, this region was reported to be a secondary HBGA binding site [16]. However, the location of this epitope on the surface of the capsid, directly proximal to the HBGA binding site, suggests that it likely plays a role in both receptor switching and in escape from herd immunity and perhaps both, simultaneously [13], [21], [40], [43]. Epitope D is close enough to the HBGA binding pocket to contribute to or inhibit carbohydrate binding, and yet variable enough to suggest that it is targeted by the immune response. Putative Epitope E is comprised of variable residues 407, 412 and 413, which are surface exposed regions lateral to the HBGA binding pockets and the other epitopes. These residues vary with every major epidemic strain after 2002, suggesting that it is a hot spot for the emergence of immunologically novel GII.4 strains. Epitope E is a GII.4.2002 blockade antibody epitope [17]. This putative epitope is lateral to the HBGA binding pockets indicating that antibodies are targeting interior regions below the capsid surface, which suggests that other epitopes may be present in the P1 subdomain. A few variable residues do not necessarily identify the boundaries of a putative epitope. Moreover, it is nearly impossible to predict the surface area of a putative epitope by sequence analysis alone. Therefore, we expanded the putative epitopes to include residues within 8 Å of the variable sites from which the epitopes were predicted (Figure 9B).

### Mapping of GII.4 blockade epitopes

**Figure 10:**
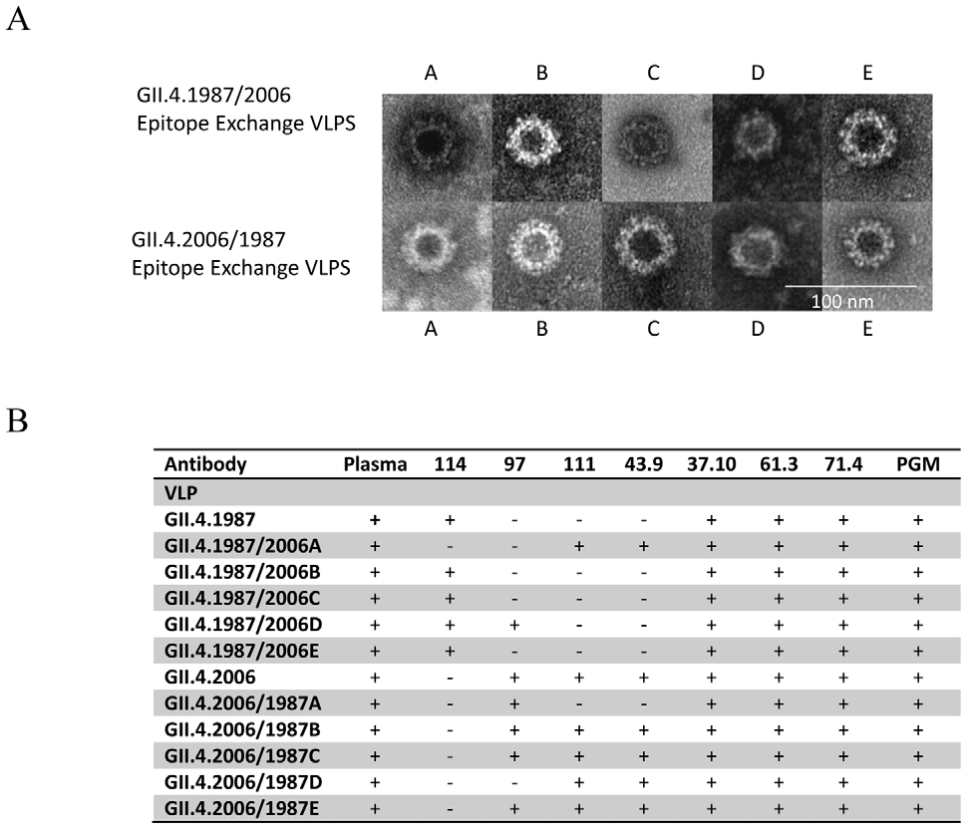
Characterization of Epitope A through E exchanged VLPs. **Panel A**: Electron microscopy visualization of negative-stained epitope-exchanged VLPs. **Panel B**: Antibody and PGM binding phenotypes of epitope-exchanged VLPs.

The described mAbs indicate at least five unique or overlapping GII.4 blockade epitopes with different specificities: 1) early GII.4 strain specific, 2) contemporary GII.4 strain specific, 3) Minerva-variant strain specific, 4) genogroup II strain specific, and 5) GII.4 strain specific. Using capsid sequences as a guide, mutant VLPs were designed to contain chimeric combinations of the predicted evolving GII.4 epitopes (Figure 10). Each predicted epitope was exchanged between the 1987 and 2006 parental strain VLPs. For example, Epitope A exchange mutant VLP GII.4.1987/2006A retains the backbone sequence of GII.4.1987 but Epitope A has been replaced with Epitope A from GII.4.2006. Whereas, GII.4.2006/1987A retains the backbone sequence of GII.4.2006, but Epitope A has been replaced with Epitope A from GII.4.1987. All epitope exchange VLPs were morphologically intact by electron microscopy visualization and retained the ability to bind PGM (Figure 10A and B, [43]), confirming chimeric VLP structural integrity.

**Figure 11:**
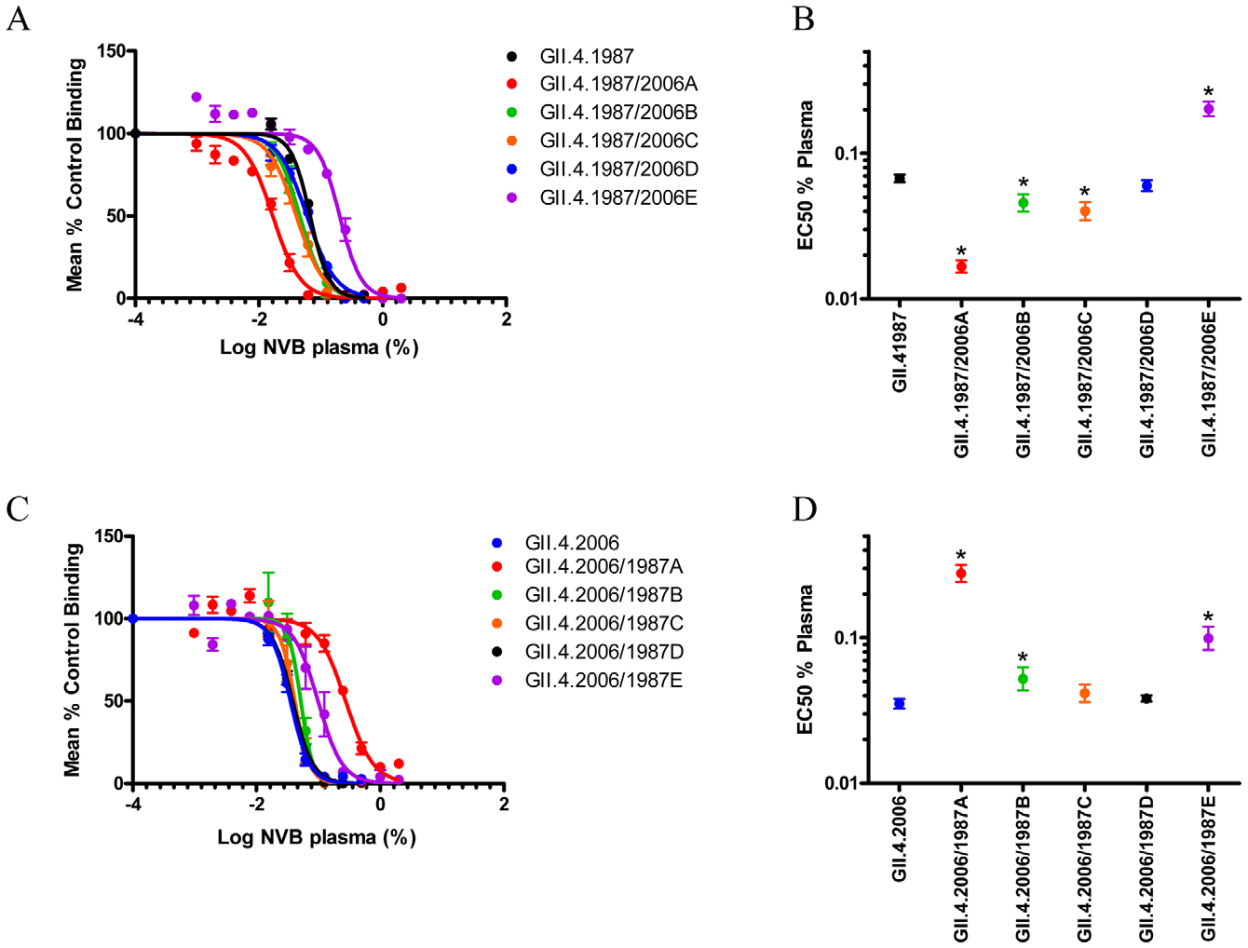
Characterization of donor NVB plasma reactivity to engineered epitope-exchanged VLPs. PGM binding blockade activity against GII.4.1987 (**Panel A**) and GII.4.2006 (**Panel C**) Epitope A–E chimeric VLPs. Sigmoidal curves were fit to the mean percent control binding calculated by comparing the amount of VLP bound to PGM in the presence of antibody pretreatment to the amount of VLP bound in the absence of antibody pretreatment. Error bars represent the standard error of the mean. Mean EC50 (% plasma) for blockade of each GII.4.1987 (**Panel B**) and GII.4.2006 (**Panel D**) epitope-exchanged VLP. * VLPs with EC50 values significantly different from the EC50 for each parental VLP.

Epitope mutant VLPs were compared to wild type strain VLPs for reactivity to the donor plasma sample. Consistent with high EIA reactivity to GII.4.1987 and 2006 VLPs (Table 2 and Figure S2), donor plasma reacted across the panel of epitope-exchange mutant VLPs (Figure 10B). Donor plasma was able to block each epitope-exchanged VLP binding to PGM (Figure 11A and C). Exchange of all of the epitopes, except Epitope D into either backbone and GII.4.1987 C into GII.4.2006 resulted in significantly different EC50 values compared to the parental strains (Figure 11B, 11D, and Table S4) (p<0.05). Only the exchange of Epitope A between the backbones resulted in an exchanged blockade phenotype, as observed with epitope-specific mAbs [43]. Exchange of Epitope A between the two parental backbones resulted in a chimeric VLP (GII.4.1987/2006A) that was blocked with significantly less plasma than the parental GII.4.1987 (EC50 0.0167 µg/ml compared to 0.0673 µg/ml) (p<0.05) and a chimeric VLP (GII.4.2006/1987A) that was blocked with significantly more plasma than the GII.4.2006 parent (EC50 0.2770 µg/ml compared to 0.0353 µg/ml) (P<0.05). In comparison, exchange of Epitope E between backbones resulted in chimeric VLPs that required significantly more antibody for blockade then either parental VLP (GII.4.1987/2006E; EC50 0.2025 µg/ml and GII.4.2006/1987E 0.0991 µg/ml) (Figure 11A–D and Table S4) (p<0.05). In this individual, these data suggest that Epitope A may be an important evolving GII.4 neutralization epitope as the blockade response is significant enough to be detected in the polyclonal antibody response.

**Figure 12:**
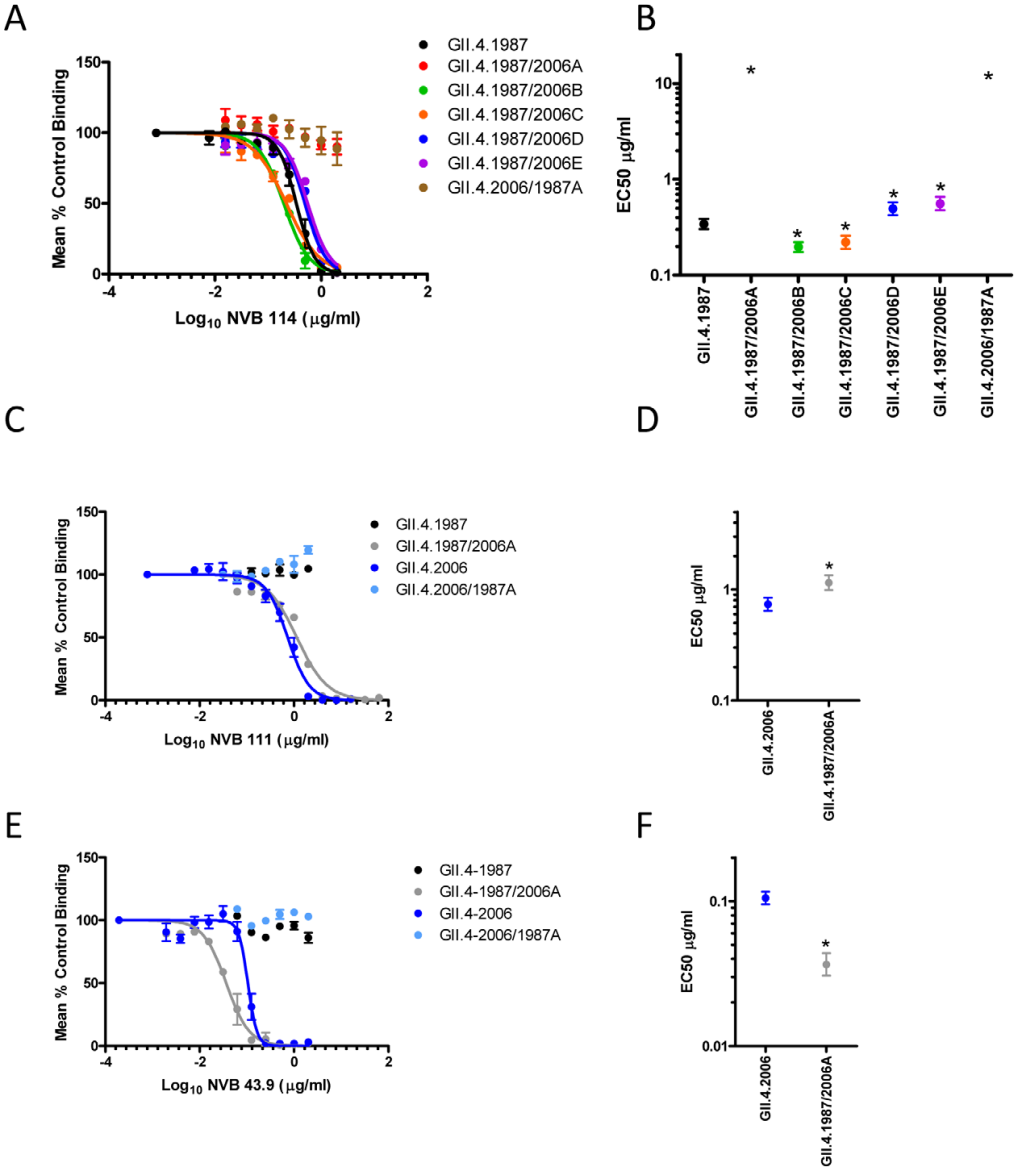
Epitope A comprises an evolving GII.4 blockade epitope recognized by NVB 114, 111 and 43.9. Hu mAb PGM binding blockade activity against GII.4.1987 epitope-exchanged VLPs for NVB 114 (**Panel A**) and Epitope A exchanged between GII.4.1987 and GII.4.2006 (**Panels C and E**). Sigmoidal curves were fit to the mean percent control binding calculated by comparing the amount of VLP bound to PGM in the presence of antibody pretreatment to the amount of VLP bound in the absence of antibody pretreatment. Error bars represent the standard error of the mean. Mean EC50 for blockade of each GII.4.1987 exchange epitope (**Panel B**) and Epitope A exchange VLPs (**Panel D and F**). * VLPs with EC50 values significantly different from the EC50 for each parental VLP.

Agreeing with the assumption that epitope-exchange mutants are unlikely to identify epitopes of cross-reactive mAbs, NVB 37.10, 61.3 and 71.4 reacted with the entire panel of chimeric VLPs by EIA (Figure 10B). In contrast, each of the strain-specific mAbs displayed differential EIA reactivity to exchanged epitopes A and D. NVB 114, 111 and 43.9 each recognized Epitope A. For NVB 114, exchange of Epitope A between the 1987 and 2006 backbones resulted in loss of antibody binding to and blockade of GII.4.1987/2006A (no blockade at 2 µg/ml) without gain of binding to GII.4.2006/1987A (Figures 12A, 10B and Table S4). Exchange of the other GII.4.1987 epitopes did not eliminate NVB 114 blockade potential (Figure 12A and B). Further, exchange of Epitope A between the 1987 and 2006 backbones resulted in loss of antibody binding to and blockade of GII.4.2006/1987A and gain of antibody binding to GII.4.1987/2006A for both NVB 43.9 and 111 (Figures 12C–F, 10B and Table S4). NVB 111 needed significantly more antibody to block GII.4.1987/2006A compared to GII.4.2006 (EC50 1.152 compared to 0.7376 µg/ml) (p<0.05) while NVB 43.9 needed slightly less antibody to block GII.4.1987/2006A compared to GII.4.2006 (EC50 0.0366 compared to 0.1031 µg/ml) (p<0.05). For both antibodies GII.4.2006/1987A was not blocked at 2 µg/ml. These data suggest that Epitope A defines a GII.4 evolving neutralization epitope for the human antibodies.

**Figure 13:**
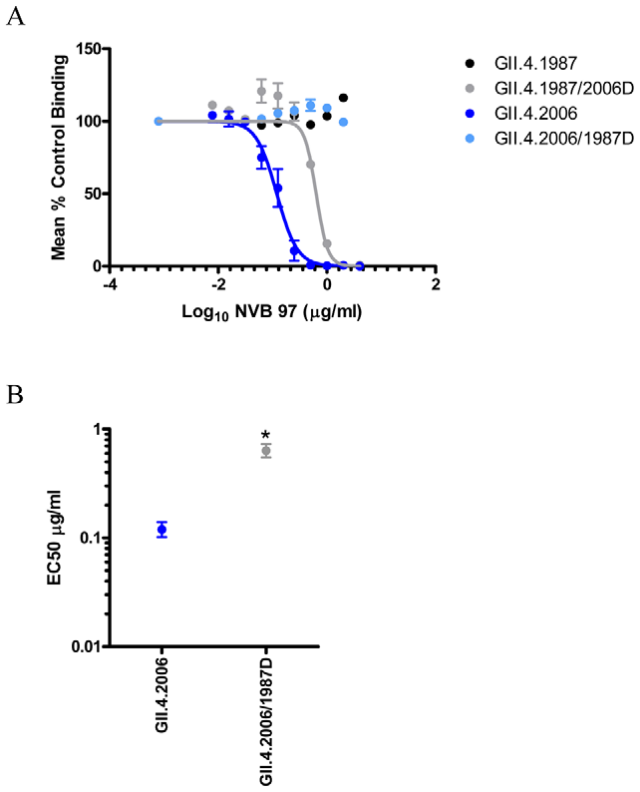
Amino acids 393–395 comprise a blockade epitope for contemporary GII.4 strains recognized by NVB 97. **Panel A**: NVB 97 PGM binding blockade activity against Epitope D exchange VLPs. Sigmoidal curves were fit to the mean percent control binding calculated by comparing the amount of VLP bound to PGM in the presence of antibody pretreatment to the amount of VLP bound in the absence of antibody pretreatment. Error bars represent the standard error of the mean. **Panel B**: Mean EC50 for blockade of each blocked VLP. * indicates VLPs with significantly different EC50 compared GII.4.2006.

**Figure 14:**
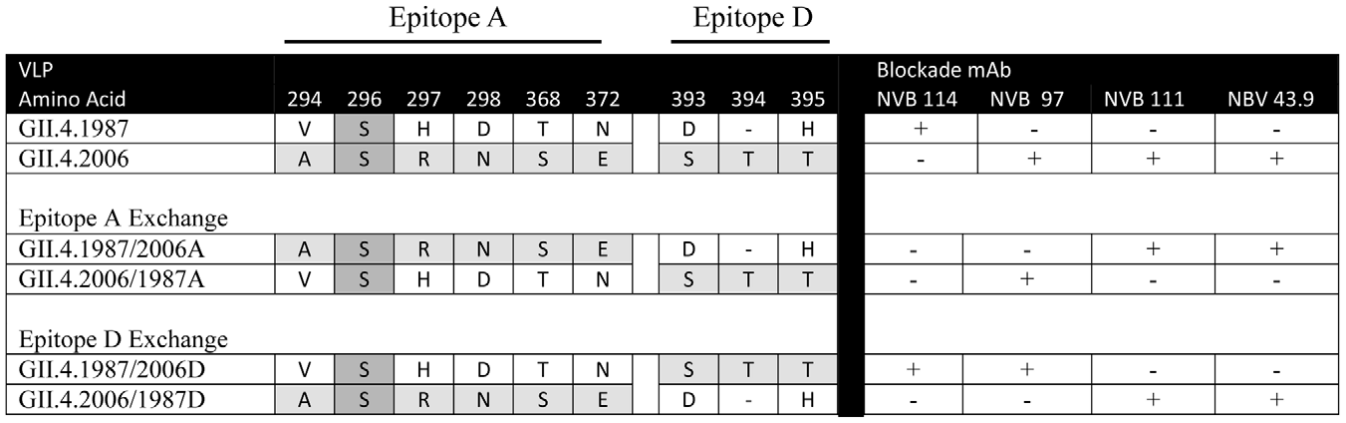
Summary of exchange mutant VLPs containing blockade Epitopes A and D and the reactivity pattern of the human mAbs that recognize these epitopes.

Similarly, the exchange of Epitope D of GII.4.2006 with Epitope D of 1987 (GII.4.2006/1987D) ablated binding of and blockade by NVB 97. Conversely, exchange of Epitope D of GII.4.1987 with Epitope D of 2006 (GII.4.1987/2006D) conferred a significant amount of binding to GII.4.1987/2006D and even blockade activity of the VLP binding to PGM (Figure 13A and B). Binding was not restored to wild type levels as the EC50 of GII.4.1987/2006D was 0.6349 µg/ml, significantly higher than the blockade EC50 for GII.4.2006 –PGM (0.1195 µg/ml) (Figure 13B and Table S4) (p<0.05). Both EIA and blockade data clearly indicate that Epitope D is critical for the binding of NVB 97 and suggest that amino acids 393–395 are important components of a GII.4 evolving blockade epitope in addition to modulating VLP-carbohydrate ligand binding [13], [21]. Interestingly, Epitope D has a single amino acid change in GII.4.2004, explaining the highly conserved binding and blockade responses noted across GII.4.2005 to 2009 VLPs, while ancestral strains display significant antigenic variation across these residues. Together, these data map the GII.4 evolving blockade epitopes recognized by each of the four strain-exclusive human mAbs described in this study (Figure 14).

## Discussion

Noroviruses are recognized as a leading cause of viral food-borne gastroenteritis. With the successful vaccination program being developed against rotavirus, focus is shifting to norovirus as the primary causative agent of severe childhood diarrhea resulting in a yearly estimate of 1.1 million episodes of pediatric gastroenteritis in developed nations and 218,000 deaths in developing nations [9]. The elderly and immunocompromised also suffer sometimes life-threatening or chronic long-term norovirus diarrheal disease characterized by malnutrition and dehydration [48]. In some HIV infected patients, chronic norovirus diarrheal disease is associated with persistent norovirus infection [49]. The economic disease burden of a norovirus outbreak within a care facility has been estimated at over $657,000 for a single event [50]. These statistics emphasize the critical need for a norovirus vaccine. Although recent reports strongly support the development and use of an efficacious norovirus vaccine in humans [38], [51], a primary obstacle to a successful vaccine is the lack of a definitive correlate to protection coupled with the extreme antigenic variation across the many norovirus strains. In fact, the existence of long-term protective immunity to norovirus infection remains controversial within the field [52]. Human challenge studies conducted before molecular diagnostics of infection and refined immune response assays had indicated that some volunteers could be reinfected with the same norovirus strain, suggesting that norovirus infection did not induce long-term protection [53]. However, recent reports identifying immune responses in norovirus-challenged but uninfected volunteers [44], [54] have necessitated qualification of these early observations to acknowledge that the findings may be compromised by assay limitations and/or the overwhelming challenge dose in comparison to the very low norovirus infectious dose [44], [54]. Clearly defining the relationships between pre-exposure history, blockade antibody responses, T cell immunity, virus evolution, and the components of protective immunity represent key challenges for future vaccine and therapeutic design.

In addition to the clinical applications of therapy and diagnosis, monoclonal antibodies have also proven to be superior tools for studying viral antigenicity, evolution, and for the treatment of acute viral disease in humans [13], [34], [55], [56]. Characterization of neutralizing mAb escape-mutants has been fundamental to identifying epitopes associated with virus receptor usage, pathogenesis, and fitness [57]–[59]. In this manuscript, we isolated and characterized the first human mAbs against noroviruses, derived from a healthy donor whose pre-exposure history was unknown. The number of unique GII.4 human monoclonal antibodies in this patient accurately reflects the high prevalence of GII.4 norovirus infection seen in human populations over the past 25 years. Moreover, distinct antibody cross reactivity patterns support the hypothesis that the GII.4 genotype is undergoing antigenic variation which not only correlates with loss of antibody blockade activity and emergence of new epidemic norovirus strains, but also changing carbohydrate ligand binding patterns over time. Importantly, antibody-mediated antigenic drift of GII.4 strains coupled with both mucosal IgA and T cell responses in challenged but uninfected volunteers strongly support the existence of long-term protective immunity against norovirus strains. In fact, the unique antibody reactivity patterns characterized herein are most likely explained as an archeological immune record of successive waves of contemporary GII.4 infections in this individual over time, implying that successful vaccine design is possible, as previously proposed by our group and others [37], [44], [54].

Previously, we have identified two immunological responses associated with protection from infection in norovirus-challenged volunteers. An early (day 1–3) post-exposure salivary IgA response in genetically susceptible volunteers correlated with protection from Norwalk virus infection [54] and an early T_h_1 response correlated with protection from Snow Mountain virus infection [44]. Historically, the role of IgG in norovirus protection has been unclear. By adulthood, >90% of the population [48] is positive for anti-norovirus IgG, and norovirus strains within a genogroup share a high degree of antigenic cross-reactivity as measured by EIA [44], [60]. These facts likely skew functional interpretations of the role of serum IgG titers on susceptibility to infection and/or infection outcomes. Although carbohydrate ligand blockade antibody responses have been suggested as correlates of protective immunity [13], [35], [36], it wasn't until recently that these blockade responses were correlated definitively with protection from clinical disease and infection [10], [38]. Importantly, our surrogate neutralization assay is specific enough to differentiate GII.4 norovirus strains too similar to be distinguished by EIA but different at key antigenic sites. While human re-challenge studies using the same viral inoculum are necessary to confirm an association between a blockade IgG response and protection from repeat norovirus infection, our findings support the clinical relevance of the antibody blockade assay as a correlate of protective immunity [38].

Monoclonal antibodies coupled with the blockade assay are powerful tools for elucidating the antigenic relationship between GII.4 strains. While mouse mAbs provide insight into GII.4 antigenic structure, data in this manuscript argues that human mAbs offer considerable advantages, including: a) immunologic record of B-cell immunity following repeat GII.4 infection, b) relationships between antibody blockade responses and antigenic variation, c) relationships between immune selection and carbohydrate ligand reactivity, d) relationships between early infection and downstream immunity, and e) epitope mapping. We focused on anti-GII.4 mAbs because of the clinical relevance of the GII.4 strains. One key finding is a direct relationship between anti-GII.4 human mAb carbohydrate ligand blockade responses and the emergence of new GII.4 epidemic strains. While similar findings were also observed for mouse anti-GII.4 mAbs, key differences were observed including the presence of human mAbs that distinguished between the 1997 and the 2002 VLPs, definitively delineating an antigenic break between the pandemic 1997 (US 95/96) and 2002 (Farmington Hills) strains. Equally important is the observation that different GII.4 antibody-blockade epitopes change with different epidemic strains. The blockade epitope recognized by NVB 114 (an early version of Epitope A) is exclusive for the early GII.4 strains, and doesn't recognize 2002 or other contemporary variant GII.4 VLPs (Figure 3). Given the lack of cross reactivity with later strains, the most likely explanation is that NVB 114 was derived from a long-term memory plasma cell that had been elicited some 12–22 years earlier. In support of this idea, examination of human monoclonal antibodies against 1918 H1N1 influenza also identified antibody variants that recognized ancestral or contemporary isolates [61]. The exclusive blockade reactivity of NVB 114 with GII.4.1987 and 1997 supports potential long-term protective immunity. Further, NVB 114 is the first antibody identified to clearly demonstrate antigenic difference between GII.4.1987 and GII.4.1997, suggesting that antigenic variation may have subtly contributed to the emergence of the GII.4 US 95/96 pandemic strain from ancestral strains.

Human monoclonal antibodies clearly identified two evolving epitopes on the surface of the GII.4 VLP. Epitopes A and D were both confirmed as evolving GII.4 antibody blockade epitopes using chimeric VLPs containing a mixture of epitopes derived from early or contemporary strains. We recognize that Epitopes A and D have not been structurally defined as epitopes, supporting the need for structural studies to define the exact mAb binding site on the VLPs. Moreover, we focused on discrete regions of varying residues and all of the residues within 8 Å (representing approximately 201 Å^2^) to the primary sites that may be influenced directly by an amino acid replacement (Figure 9B). However, Ab binding sites have been reported to be much larger (700–800 Å^2^), suggesting that the epitopes that we have predicted may actually work in concert to form larger Ab recognition sites. By expanding the putative epitopes, we identified other residues that were less variable; however, the exact role that these varying residues play in evolution is less clear. In some cases variation may be required to encode changes that are necessary for the replacement at a primary site. In addition, all of our structural analyses have been conducted using models of the P dimer, representing about 1/90^th^ of the VLP structural surface. Interactions between the epitopes in the context of the superstructure have not been determined. Therefore, these observations indicate that the complex nature of the NoV Ab epitope requires further research to define the specific boundaries and residues that regulate Ab binding.

The prediction of five putative epitopes allowed us to gain several important insights into GII.4 norovirus evolution: 1) Discrete sites of variation occur on the GII.4 norovirus capsid, either directly on the surface or lateral to the HBGA binding sites; 2) Secondary variable sites are within 8 Å of the primary variable sites, and these secondary sites could also contribute to epitope remodeling; 3) Many of the putative expanded epitopes overlap, suggesting that two or more highly variable epitopes may work in concert to escape from an antibody response; 4) Putative epitopes that are buried may exert an effect on the structure by altering the interior fold space, allowing unconventional replacements to be tolerated; 5) An underlying amino acid network likely preserves the functional core of the capsid proteins by regulating the variable residues above them; and 6) Escape and HBGA binding may be intimately linked via the underlying regulatory network of amino acids that preserve the functional integrity of the capsid core.

Epitope A, which likely includes varying amino acid residues 294, 296–298 and 368 and 372 and potentially other undefined nearby residues, has been mapped as a blockade epitope in both GII.4.1987 and GII.4.2006 Minerva variants. Importantly, the antibodies that recognize Epitope A in GII.4.1987 do not bind GII.4.2006 and the antibodies that recognize Epitope A in GII.4.2006 did not recognize GII.4.1987 (Figures 3, 5, 12 and [43]). These data support the idea that unique memory B cells were elicited as a consequence of unique exposure events, most likely years apart in this individual. Further, the significant difference in EC50 values between the 2006 and 2009 Minerva variants for NVB 111 and 43.9 (Figure 5) suggests that Epitope A continues to evolve and the epidemic 2009 stain is still diverging from the pandemic 2006 strain at this site, most likely as a consequence of long-term antibody selection. GII.4.2006 and 2009 differ at positions 294, 368 and 372 within Epitope A (Figure 9A). At this time it is unclear how many and which amino acids in Epitope A are needed to mediate an escape mutant phenotype that is completely resistant to GII.4.2006 antibody blockade. However, it is clear that Epitope A varies, and that the site is conserved as a major target for blockade antibody response between 1987 and 2009. Although correlative, comparisons of Epitope A variation along with residues that are proximal to those that appear to be evolving over time suggest that changes at positions 292, 293, 294, 295, 296, 297, 298, 300, 365, 367, 368 and 372 might contribute to an escape phenotype, with residues 294, 296, 297, 298, 300, 368 and 372 playing direct roles in this variation (Figure 9). However, the minor replacements at other positions are likely essential for remodeling the local structural neighborhood such that more profound changes can be tolerated. Supporting the sensitivity of epitopes to the local environment, mAbs that recognized Epitope A differentiated between GII.4.2006 VLPs and GII.4.2006 P dimers. P protein (P dimer) is a dimeric, truncated form of the major capsid protein composed of residues 214–539 [62]. P-dimers have been widely used to determine the crystallographic structure of NoV-HBGA interactions and are considered accurate reflections of the VLP surface topology [16], [21], [63]. P –particles can assemble as higher ordered structures composed of varying copies of the P dimer [46], [64]. These subviral particles are reported to have similar characteristics to VLPs [62], [65]–[67] and have been proposed as a candidate vaccine platforms [68]. To our knowledge, this is the first immunologic characterization of P-dimers vs. VLPs using monoclonal antibodies derived from human infections. Here, P-dimers derived from GII.4.2006 lost binding of mAbs NVB 111 and 43.9, the mAbs that recognize Epitope A in GII.4.2006. P dimer binding to the Epitope D binding mAb NVB 97 and the broadly cross-blockade mAb NVB 71.4 were retained. Recently, P-particle vaccines were shown to be less robust at inducing strong blockade responses, as compared with intact VLP [69], perhaps because of the loss of blockade Epitope A in this higher ordered structure. While speculative, it is recognized that virions “breathe” suggesting that the possibility that P-dimers and P-particles may become “locked” in a slightly less immunologically reactive state that affects some but not all blockade epitopes on the virion surface [70], [71]. These data absolutely underscore the critical importance of determining the structures of several of these human mAbs with their appropriate GII.4 VLP epitopes by either cryoEM or crystallography, for informing targeted mutagenesis to identify the role of key residues in regulating antigenicity and antibody escape.

Epitope D is a conformational epitope comprised of varying amino acids 393–395 and likely other nearby residues that are less clearly defined; however, additional mapping and crystallographic studies will be needed to clarify this epitope structure. With the emergence of the pandemic GII.4.2004 Hunter strain, Epitope D elicited robust antibody blockade responses as typified by NVB 97. These observations suggest that Epitope D has been relatively static since 2004 and may be a good target for vaccine development. Although the blockade epitope is conserved post 2002, comparison of EC50 titers suggests that the antibody's highest efficacy is against GII.4.2006, as all other VLPs have significantly less robust EC50 values (Figure 4). Previously, these residues have been implicated in regulating norovirus VLP-carbohydrate ligand binding interactions [13], [21], [43]. The identification of Epitope D as a human antibody blockade epitope that changes over time provides direct support for our previous hypothesis that escape from protective herd immunity may drive changes in carbohydrate ligand binding affinities over time and potential retargeting of virus infection in different human populations [13], [40].

These data indicate that like influenza, a successful norovirus vaccine regimen will require periodic population sampling to identify future strains for inclusion into the next year's vaccine formulation not unlike the strategy employed by the Influenza Virus Global Surveillance Program. Norovirus population sampling has already begun as monitoring systems for detection of NoV infections have been established in the United States, Europe, and Japan. Identification of GII.4 evolving antigenic epitopes furthers our understanding of norovirus pathogenesis and provides target epitopes that may be useful for surveillance and prediction of new strain emergence. Identification of GII.4 conserved epitopes also informs diagnostic and potential therapeutic reagent development and design. Monoclonal antibodies NVB 37.10, 61.3 and 71.4 all recognize epitopes conserved among the GII.4 strains from 1987 through 2009. NVB 37.10 and 61.3 have enhanced GII VLP recognition, binding not only GII.4 VLPs but also other GII VLPs, but are unable to block VLP-PGM interaction. The high conservation of the NVB 37.10 and 61.3 epitopes suggest that these epitopes are highly resistant to antigenic variation within the GII strains, making these mAbs potentially valuable diagnostic reagents as GII strains cause up to 95% of norovirus outbreaks [27], [72]. The unidentified epitope for NVB 71.4 is clearly different from the epitopes recognized by NVB 37.10 and 61.3 and is conserved throughout the GII.4 strains. NVB 71.4 did not recognize any non-GII.4 VLPs but, importantly, it exhibited blockade activity for the entire panel of time-ordered GII.4 VLPs with PGM and Bi-HBGAs. Emphasizing the difference between the two quantitative blockade assays and the qualitative HAI assay, GII.4.2002 HA was not inhibited by NVB 71.4. Noting that blockade assays are not true measurements of neutralization, NVB 71.4 has potential as a therapeutic reagent based on its broad GII.4 blockade potential and the fact that it is by nature a human antibody. Clearly, the effectiveness of NVB 71.4 at preventing or treating illness can only be determined empirically. Although one of the blockade-epitope specific mAbs with lower EC50 values/steeper Hill constants may be more effective at select strain neutralization, the breadth of strains neutralized is likely to be limited for these mAbs. There are a number of viral diseases currently being treated with mAbs including RSV, CMV and enterovirus; however, only the anti-RSV humanized mAb palivizumab has FDA approval for prophylactic use in humans [73], [74]. In outbreak settings or in chronically infected patients, an anti-NoV mAb that could be delivered before symptoms begin and protect from illness could be very useful in care facilities, the military and the cruise industry. Given the acute clinical disease window, it is less likely that therapeutic antibodies will provide relief in those individuals experiencing acute infections, however, therapeutic antibodies may offer opportunities for ameliorating symptomatic disease in chronic infections. The discovery of broadly cross reactive and cross blockade human GII.4 mAbs dictates the need for a new approach to map epitopes. Our current experimental approach was designed to identify GII.4 epitopes that change over time and provide insight into broadly conserved epitope locations. Because the identification of the epitopes recognized by NVB 37.10, 61.3, and 71.4 has important implications for successful vaccine design, new panels of mutated VLPs and other approaches will be needed to characterize these epitopes in the future.

GII.4 NoVs are significant human pathogens that cause considerable morbidity and mortality, worldwide. The development of mouse mAbs to different time-ordered GII.4 VLPs has greatly facilitated progress towards understanding the complex antigenic relationships between these strains by clearly demonstrating antigenic variation over time and epidemic strain [34]. Here we have expanded these observations using human anti-GII.4 mAbs isolated from a healthy adult donor, who has likely experienced multiple norovirus infections throughout his/her lifetime. The identification of highly significant, varying antigenic epitopes that influence VLP-carbohydrate ligand interaction provides important new insights into vaccine design and the development of therapeutics that target norovirus virions. For example, these antibodies represent the first anti-norovirus human mAbs to be characterized, and they confirm findings from studies using mouse mAbs supporting antigenic drift and its linkage with varying carbohydrate ligand binding profiles within the GII.4 noroviruses. Further, we have demonstrated that the GII.4 NoV varying epitopes can be exchanged between time-ordered VLPs, providing a robust platform for expanding the antigenic and blockade cross reactivity of future vaccine candidates. Using this approach, we have identified two surface-exposed antibody blockade epitopes that vary over time and were differentially recognized by four of the seven human mAbs. We also identified three antibodies which recognize either overlapping or three unique highly conserved epitopes within the GII.4 VLP. These data continue to support the hypothesis that norovirus long-term protective immune responses are elicited following acute infection, a concept essential for effective vaccine design. We anticipate that a full understanding of the varying antigenic and blockade epitopes of GII.4 NoVs may not only help to predict the emergence of new epidemic strains but simultaneously identify key reformulations in vaccine design that will protect public health against contemporary and emerging epidemic strains in the future.

## Materials and Methods

### Virus-like particles (VLPs)

A diverse panel of VLPs representing G1 and GII norovirus strains and epitope mutants was assembled as previously reported [13], [17], [75]. To design epitope exchange chimeric VLPs, we first identified surface exposed residue clusters that varied over time. Then, we synthesized a series of chimeric GII.4 ORF2 genes that exchanged “putative” epitopes between GII.4.1987 and GII.4.2006 VLPs [43]. For all constructs except GII.4.2009 ORF2 [17], the synthetically derived constructs were inserted directly into the VEE replicon vector for the production of virus replicon particles (VRPs) as previously described by our group. VLPs were expressed in VRP-infected BHK cells and purified by velocity sedimentation in sucrose and stored at −80°C. The GII.4.2009 (New Orleans [17]) VLPs were expressed in the baculovirus system and purified by cesium chloride gradient centrifugation and were the kind gift of Dr. Jan Vinje, Centers for Disease Control and Prevention, Atlanta, GA. VLP protein concentrations were determined by the BCA Protein Assay (Pierce, Rockford, IL). VLP preparation purity averaged >80% by SDS-Page analysis.

### Human monoclonal antibody production

In early 2009, following written consent, blood samples from 63 donors were collected from adult healthy donors at the Lugano and Basel Blood banks (Switzerland). Peripheral blood mononuclear cells (PBMCs) and plasma were isolated and cryopreserved. On the day of use, PBMCs from Donor 302898 (Figure 1), an individual born in 1948, were thawed and IgG^+^ memory B cells were isolated using CD22 microbeads (Miltenyi) followed by cell sorting, as described [76]. Cells were immortalized at 5 cells/well in multiple cultures using EBV in the presence of CpG oligodeoxynucleotide 2006 (Microsynth) and irradiated allogeneic PBMC. After 2 weeks, culture supernatants were screened for the presence of norovirus-specific mAbs by EIA against VLPs and positive cultures were cloned by limiting dilution. Antibodies were recovered from supernatants and purified using protein A affinity chromatography and finally desalted against PBS using a HiTrap FastDesalting column.

### EIAs

Human mAb reactivity was determined by EIA, as reported [34]. Briefly, plates were coated at 1 µg/ml VLP in PBS before the addition 1 µg/ml purified IgG or donor plasma (0.2%). Primary antibody incubation was followed by anti-human IgG-alkaline phosphatase and color development with pNPP substrate solution (Sigma Chemicals, St. Louis, MO). Each step was followed by washing with PBS-0.05% Tween-20 and all antibodies were diluted in 5% dry milk in PBS-0.05% Tween-20. Data shown represent the average of at least three replicates and are representative of similar data from at least two independent trials. Establishment of EIAs using new mAbs included PBS-coated wells as negative controls and polyclonal anti-norovirus human sera as positive controls. Antibodies were considered positive for reactivity if the mean optical density after background subtraction for VLP-coated wells was greater than three times the mean optical density for PBS-coated wells [34]. For screening donor plasma samples, the binding titers of plasma to respective coated VLPs were determined by EIA as described above by measuring the plasma dilution required to achieve 50% maximal binding (ED50). EIA reactivity to GII.4.2006 P protein (amino acids 221–531 [21]) was measured similarly to reactivity to VLP. GII.4.2006 P protein was the kind gift of B.V. Prasad, Baylor College of Medicine, Houston, TX).

### VLP-Carbohydrate ligand-binding antibody blockade assays

Pig Gastric Mucin Type III (PGM) (Sigma Chemicals) has been validated as a substrate for NoV VLP antibody-blockade assays [17]. PGM contains relatively high levels of H and A antigen and more moderate levels of Lewis Y antigen [17]. All of the GII.4 VLPs used in the blockade assays in this study bind to both PGM and synthetic Bi-HBGA, and binding to PGM is consistent with synthetic Bi-HBGA binding profiles for α-1,2-fucose (H antigen) and α-1,4-fucose (Lewis antigen) containing molecules [13], [17], [77]. For blockade assays, PGM was solvated in PBS at 5 mg/ml and coated onto EIA plates at 10 µg/ml in PBS for 4 hours and blocked over night at 4°C in 5% dry milk in PBS-0.05% Tween-20. VLPs (0.5 µg/ml) were pretreated with decreasing concentrations of test mAb or donor plasma for 1 hour at room temperature before being added to the carbohydrate ligand–coated plates for 1 hour. Bound VLP was detected by a rabbit anti-GII norovirus polyclonal sera made from hyperimmunization with either GII.4.2009 or a cocktail of GII.4.1997, GII.3.1999, GII.1.1976, and GII.2.1976 VLPs, followed by anti-rabbit IgG-HRP (GE Healthcare) and color developed with 1-Step Ultra TMB ELISA HRP substrate solution (Thermo-Fisher). The percent control binding was defined as the binding level in the presence of antibody pretreatment compared to the binding level in the absence of antibody pretreatment multiplied by 100. All incubations were done at room temperature. Each step was followed by washing with PBS-0.05% Tween 20 and all reagents were diluted in 5% dry milk in PBS-0.05% Tween-20. All antibodies were tested for blockade potential against the panel of GII.4 VLPs at two-fold serial dilutions ranging from 0.08 to 2 µg/ml. Additional concentrations of blockade antibodies were tested if needed to complete the sigmoid dose-response curve. Blockade of synthetic Bi-HBGAs (Glycotech, Gaithersburg, MD) assays were done as described for PGM with the following exception. Bi-HBGAs were bound to Neutri-avidin coated plates (Pierce) at 10 µg/ml for one hour prior to the addition of 1 µg/ml VLP for 1.5 h. Reported mean % control binding reflects the results of at least two independent experiments with each dilution tested at least in duplicate. An antibody was designated as a “blockade” antibody for a VLP if at least 50% of control binding was inhibited by 2 µg/ml antibody. Blockade data were fitted and EC50 values calculated using Sigmoidal dose response analysis of non-linear data in GraphPad Prism 5 (www.graphpad.com). EC50 values between VLPs were compared using the One-way ANOVA with Dunnett post test, when at least three values were compared or the unpaired t-test when two values were compared. A difference was considered significant if the P value was <0.05. To test for antibody binding that prevents detector antibody from binding to the VLP instead of the VLP binding to the PGM, select blockade assays are performed without using a detector antibody and instead developed directly with an anti-human IgG-HRP. Antibodies tested this way give two responses; 1) a bell-shaped response curve for antibodies that are blockade and 2) a sigmoidal shaped curve for antibodies that are not blockade. These data indicate that it is the amount of human mAb that is directly blocking the VLP from binding to PGM. Of note, VLP concentrations in blockade assays are in the low nanomolar range and therefore cannot discriminate between antibodies with sub-nanomolar affinities.

### Hemagglutination inhibition assays

HAI assays were performed as reported [38], [39], [45]. VLPs at 50 ng/reaction were pretreated with antibody as described above for the blockade assays before addition to O+ RBCs at 4°C, pH 5.5. An HAI titer was determined as the lowest antibody concentration that completely prevented NoV VLP-induced HA by visualization.

### Structural models of VLP P domains

The amino acid sequences of GII.4.1987, GII.4.2002, and GII.4.2006 capsids were individually aligned to the VA387 P domain sequence using Clustalx1.86 [78], and the GII.4.2002 domain dimer X-ray crystal structure (PDB accession: 2OBT) [16] was used as a template for generating homology models. Homology models were generated using the program Modeller available via the Max Planck Institute Bioinformatics Toolkit (http://toolkit.tuebingen.mpg.de/). The structural models were analyzed and compared, and figures were generated using Mac Pymol (Delano Scientific).

## Supporting Information

Figure S1 **NoV VLP EC50 (reciprocal dilution) in individual plasma samples by VLP.** EIA reactivity to individual VLPs is color coded: White; EC50 dilution <100, Purple; EC50 dilution between 100–500, Blue; EC50 dilution between 501–1999, Pink; EC50 dilution ≥2000. Monoclonal antibodies were developed from Donor 302898, named NVB.
(PDF)

Figure S2 **EIA Reactivity of NVB plasma and mAbs against NoV VLPs.** Columns present the mean OD of 0.2% NVB plasma or 1 µg/ml mAb reactivity with immobilized VLP. **Panel A**: Antibodies with broad genogroup II VLP reactivity. **Panel B**. Antibodies with restricted GII.4 VLP reactivity. Bars are SEM. Mean ODs above 3-fold background (dashed line) were scored as positive.
(TIF)

Table S1 **Antibody EC50 µg/ml (95% CI) blockade of VLP binding to PGM.**
(DOC)

Table S2 **Antibody EC50 µg/ml (95% CI) blockade of VLP binding to synthetic biotinylated HBGAs.**
(DOCX)

Table S3 **NVB plasma (%) and monoclonal antibody (µg/ml) HAI titer.**
(DOC)

Table S4 **Plasma (%) and Antibody (µg/ml) EC50 (95% CI) blockade of epitope exchanged VLP binding to PGM.**
(DOC)

## References

[1] (2011). Updated norovirus outbreak management and disease prevention guidelines.. MMWR Recomm Rep.

[2] Hutson AM, Atmar RL, Estes MK (2004). Norovirus disease: changing epidemiology and host susceptibility factors.. Trends Microbiol.

[3] Estes MK, Prasad BV, Atmar RL (2006). Noroviruses everywhere: has something changed?. Curr Opin Infect Dis.

[4] Koopmans M, Vinj inverted question marke J, de Wit M, Leenen I, van der Poel W (2000). Molecular epidemiology of human enteric caliciviruses in The Netherlands.. J Infect Dis.

[5] (2007). Norovirus activity–United States, 2006–2007.. MMWR Morb Mortal Wkly Rep.

[6] Okada M, Tanaka T, Oseto M, Takeda N, Shinozaki K (2006). Genetic analysis of noroviruses associated with fatalities in healthcare facilities.. Arch Virol.

[7] Harris JP, Edmunds WJ, Pebody R, Brown DW, Lopman BA (2008). Deaths from norovirus among the elderly, England and Wales.. Emerg Infect Dis.

[8] Schorn R, Hohne M, Meerbach A, Bossart W, Wuthrich RP (2010). Chronic norovirus infection after kidney transplantation: molecular evidence for immune-driven viral evolution.. Clin Infect Dis.

[9] Patel MM, Widdowson MA, Glass RI, Akazawa K, Vinje J (2008). Systematic literature review of role of noroviruses in sporadic gastroenteritis.. Emerg Infect Dis.

[10] Atmar RL, Bernstein DI, Harro CD, Al-Ibrahim MS, Chen WH, Ferreria J, Estes MK, Graham DY, Opekun AR, Richardson C, Mendelman PM (2011). Norovirus Vaccine against Experiemntal Human Norwalk Virus Illness.. N Engl J Med.

[11] Prasad BV, Hardy ME, Dokland T, Bella J, Rossmann MG (1999). X-ray crystallographic structure of the Norwalk virus capsid.. Science.

[12] Zheng DP, Ando T, Fankhauser RL, Beard RS, Glass RI (2006). Norovirus classification and proposed strain nomenclature.. Virology.

[13] Lindesmith LC, Donaldson EF, Lobue AD, Cannon JL, Zheng DP (2008). Mechanisms of GII.4 norovirus persistence in human populations.. PLoS Med.

[14] Chen R, Neill JD, Estes MK, Prasad BV (2006). X-ray structure of a native calicivirus: structural insights into antigenic diversity and host specificity.. Proc Natl Acad Sci U S A.

[15] Lochridge VP, Jutila KL, Graff JW, Hardy ME (2005). Epitopes in the P2 domain of norovirus VP1 recognized by monoclonal antibodies that block cell interactions.. J Gen Virol.

[16] Cao S, Lou Z, Tan M, Chen Y, Liu Y (2007). Structural Basis for the Recognition of Blood Group Trisaccharides by Norovirus.. J Virol.

[17] Lindesmith LC, Debbink K, Swanstrom J, Vinje J, Costantini V (2012). Monoclonal antibody-based antigenic mapping of norovirus GII.4-2002.. J Virol.

[18] Siebenga JJ, Vennema H, Renckens B, de Bruin E, van der Veer B (2007). Epochal Evolution of GGII.4 Norovirus Capsid Proteins from 1995 to 2006.. J Virol.

[19] Bull RA, Eden JS, Rawlinson WD, White PA (2010). Rapid evolution of pandemic noroviruses of the GII.4 lineage.. PLoS Pathog.

[20] Bok K, Abente EJ, Realpe-Quintero M, Mitra T, Sosnovtsev SV (2009). Evolutionary dynamics of GII.4 noroviruses over a 34-year period.. J Virol.

[21] Shanker S, Choi JM, Sankaran B, Atmar RL, Estes MK (2011). Structural Analysis of HBGA Binding Specificity in a Norovirus GII.4 Epidemic Variant: Implications for Epochal Evolution.. J Virol.

[22] Allen DJ, Gray JJ, Gallimore CI, Xerry J, Iturriza-Gomara M (2008). Analysis of amino acid variation in the P2 domain of the GII-4 norovirus VP1 protein reveals putative variant-specific epitopes.. PLoS ONE.

[23] de Rougemont A, Ruvoen-Clouet N, Simon B, Estienney M, Elie-Caille C (2011). Qualitative and quantitative analysis of the binding of GII.4 norovirus variants onto human blood group antigens.. J Virol.

[24] Noel JS, Fankhauser RL, Ando T, Monroe SS, Glass RI (1999). Identification of a distinct common strain of “Norwalk-like viruses” having a global distribution.. J Infect Dis.

[25] Vinje J, Altena S, Koopmans M (1997). The incidence and genetic variability of small round-structured viruses in outbreaks of gastroenteritis in the Netherlands.. J Infect Dis.

[26] Widdowson MA, Cramer EH, Hadley L, Bresee JS, Beard RS (2004). Outbreaks of acute gastroenteritis on cruise ships and on land: identification of a predominant circulating strain of norovirus–United States, 2002.. J Infect Dis.

[27] Fankhauser RL, Monroe SS, Noel JS, Humphrey CD, Bresee JS (2002). Epidemiologic and molecular trends of “Norwalk-like viruses” associated with outbreaks of gastroenteritis in the United States.. J Infect Dis.

[28] Bull RA, Tu ET, McIver CJ, Rawlinson WD, White PA (2006). Emergence of a new norovirus genotype II.4 variant associated with global outbreaks of gastroenteritis.. J Clin Microbiol.

[29] Kroneman A, Vennema H, Harris J, Reuter G, von Bonsdorff CH (2006). Increase in norovirus activity reported in Europe.. Euro Surveill.

[30] Phan TG, Kuroiwa T, Kaneshi K, Ueda Y, Nakaya S (2006). Changing distribution of norovirus genotypes and genetic analysis of recombinant GIIb among infants and children with diarrhea in Japan.. J Med Virol.

[31] Siebenga J, Kroneman A, Vennema H, Duizer E, Koopmans M (2008). Food-borne viruses in Europe network report: the norovirus GII.4 2006b (for US named Minerva-like, for Japan Kobe034-like, for UK V6) variant now dominant in early seasonal surveillance.. Euro Surveill.

[32] Vega E BL, Gregoricus N, Williams K, Lee D, Vinjé J (2011). Novel surveillance network for norovirus gastroenteritis outbreaks, United States.. Emerg Infect Dis.

[33] Cannon JL, Lindesmith LC, Donaldson EF, Saxe L, Baric RS (2009). Herd immunity to GII.4 noroviruses is supported by outbreak patient sera.. J Virol.

[34] Lindesmith LC, Donaldson EF, Baric RS (2011). Norovirus GII.4 strain antigenic variation.. J Virol.

[35] Harrington PR, Lindesmith L, Yount B, Moe CL, Baric RS (2002). Binding of Norwalk virus-like particles to ABH histo-blood group antigens is blocked by antisera from infected human volunteers or experimentally vaccinated mice.. J Virol.

[36] Lindesmith LC, Donaldson E, Leon J, Moe CL, Frelinger JA (2010). Heterotypic humoral and cellular immune responses following Norwalk virus infection.. J Virol.

[37] Bok K, Parra GI, Mitra T, Abente E, Shaver CK (2011). Chimpanzees as an animal model for human norovirus infection and vaccine development.. Proc Natl Acad Sci U S A.

[38] Reeck A, Kavanagh O, Estes MK, Opekun AR, Gilger MA (2010). Serological Correlate of Protection against Norovirus-Induced Gastroenteritis.. J Infect Dis.

[39] Czako R, Atmar RL, Opekun AR, Gilger MA, Graham DY (2012). Serum hemagglutination inhibition activity correlates with protection from gastroenteritis in persons infected with Norwalk virus.. Clin Vaccine Immunol.

[40] Donaldson EF, Lindesmith LC, Lobue AD, Baric RS (2008). Norovirus pathogenesis: mechanisms of persistence and immune evasion in human populations.. Immunol Rev.

[41] Siebenga JJ, Lemey P, Kosakovsky Pond SL, Rambaut A, Vennema H (2010). Phylodynamic reconstruction reveals norovirus GII.4 epidemic expansions and their molecular determinants.. PLoS Pathog.

[42] Allen DJ, Noad R, Samuel D, Gray JJ, Roy P (2009). Characterisation of a GII-4 norovirus variant-specific surface-exposed site involved in antibody binding.. Virol J.

[43] Debbink K, Donaldson EF, Lindesmith LC, Baric RS (2011). Genetic Mapping of a Highly Variable Norovirus GII.4 Blockade Epitope: Potential Role in Contribution in Escape from Human Herd Immunity.. J Virol.

[44] Lindesmith L, Moe C, Lependu J, Frelinger JA, Treanor J (2005). Cellular and humoral immunity following Snow Mountain virus challenge.. J Virol.

[45] Hutson AM, Atmar RL, Marcus DM, Estes MK (2003). Norwalk virus-like particle hemagglutination by binding to h histo-blood group antigens.. J Virol.

[46] Tan M, Jiang X (2005). The p domain of norovirus capsid protein forms a subviral particle that binds to histo-blood group antigen receptors.. J Virol.

[47] Tan M, Xia M, Cao S, Huang P, Farkas T (2008). Elucidation of strain-specific interaction of a GII-4 norovirus with HBGA receptors by site-directed mutagenesis study.. Virology.

[48] Koo HL, Ajami N, Atmar RL, DuPont HL (2010). Noroviruses: The leading cause of gastroenteritis worldwide.. Discov Med.

[49] Wingfield T, Gallimore CI, Xerry J, Gray JJ, Klapper P (2010). Chronic norovirus infection in an HIV-positive patient with persistent diarrhoea: a novel cause.. J Clin Virol.

[50] Johnston CP, Qiu H, Ticehurst JR, Dickson C, Rosenbaum P (2007). Outbreak management and implications of a nosocomial norovirus outbreak.. Clin Infect Dis.

[51] El-Kamary SS, Pasetti MF, Mendelman PM, Frey SE, Bernstein DI (2010). Adjuvanted intranasal norwalk virus-like particle vaccine elicits antibodies and antibody-secreting cells that express homing receptors for mucosal and peripheral lymphoid tissues.. The J Infect Dis.

[52] Glass RI, Parashar UD, Estes MK (2009). Norovirus gastroenteritis.. N Engl J Med.

[53] Parrino TA, Schreiber DS, Trier JS, Kapikian AZ, Blacklow NR (1977). Clinical immunity in acute gastroenteritis caused by Norwalk agent.. N Engl J Med.

[54] Lindesmith L, Moe C, Marionneau S, Ruvoen N, Jiang X (2003). Human susceptibility and resistance to Norwalk virus infection.. Nat Med.

[55] Saylor C, Dadachova E, Casadevall A (2009). Monoclonal antibody-based therapies for microbial diseases.. Vaccine.

[56] Nelson AL, Dhimolea E, Reichert JM (2010). Development trends for human monoclonal antibody therapeutics.. Nat Rev Drug Discov.

[57] Rockx B, Donaldson E, Frieman M, Sheahan T, Corti D (2010). Escape from human monoclonal antibody neutralization affects in vitro and in vivo fitness of severe acute respiratory syndrome coronavirus.. J Infect Dis.

[58] Kaufmann B, Vogt MR, Goudsmit J, Holdaway HA, Aksyuk AA (2010). Neutralization of West Nile virus by cross-linking of its surface proteins with Fab fragments of the human monoclonal antibody CR4354.. Proc Natl Acad Sci U S A.

[59] Whittle JR, Zhang R, Khurana S, King LR, Manischewitz J (2011). Broadly neutralizing human antibody that recognizes the receptor-binding pocket of influenza virus hemagglutinin.. Proc Natl Acad Sci U S A.

[60] Noel JS, Ando T, Leite JP, Green KY, Dingle KE (1997). Correlation of patient immune responses with genetically characterized small round-structured viruses involved in outbreaks of nonbacterial acute gastroenteritis in the United States, 1990 to 1995.. J Med Virol.

[61] Yu X, Tsibane T, McGraw PA, House FS, Keefer CJ (2008). Neutralizing antibodies derived from the B cells of 1918 influenza pandemic survivors.. Nature.

[62] Tan M, Hegde RS, Jiang X (2004). The P domain of norovirus capsid protein forms dimer and binds to histo-blood group antigen receptors.. J Virol.

[63] Choi JM, Hutson AM, Estes MK, Prasad BV (2008). Atomic resolution structural characterization of recognition of histo-blood group antigens by Norwalk virus.. Proc Natl Acad Sci U S A.

[64] Bereszczak JZ, Barbu IM, Tan M, Xia M, Jiang X (2012). Structure, stability and dynamics of norovirus P domain derived protein complexes studied by native mass spectrometry.. J Struct Biol.

[65] Tan M, Fang PA, Xia M, Chachiyo T, Jiang W (2011). Terminal modifications of norovirus P domain resulted in a new type of subviral particles, the small P particles.. Virology.

[66] Tan M, Fang P, Chachiyo T, Xia M, Huang P (2008). Noroviral P particle: structure, function and applications in virus-host interaction.. Virology.

[67] Tan M, Zhong W, Song D, Thornton S, Jiang X (2004). E. coli-expressed recombinant norovirus capsid proteins maintain authentic antigenicity and receptor binding capability.. J Med Virol.

[68] Tan M, Huang P, Xia M, Fang PA, Zhong W (2011). Norovirus P particle, a novel platform for vaccine development and antibody production.. J Virol.

[69] Tamminen K, Huhti L, Koho T, Lappalainen S, Hytonen VP (2012). A comparison of immunogenicity of norovirus GII-4 virus-like particles and P-particles.. Immunology.

[70] Pierson TC, Fremont DH, Kuhn RJ, Diamond MS (2008). Structural insights into the mechanisms of antibody-mediated neutralization of flavivirus infection: implications for vaccine development.. Cell Host Microbe.

[71] Nelson S, Jost CA, Xu Q, Ess J, Martin JE (2008). Maturation of West Nile virus modulates sensitivity to antibody-mediated neutralization.. PLoS Pathog.

[72] Lopman B, Vennema H, Kohli E, Pothier P, Sanchez A (2004). Increase in viral gastroenteritis outbreaks in Europe and epidemic spread of new norovirus variant.. Lancet.

[73] Zhu Z, Dimitrov AS, Chakraborti S, Dimitrova D, Xiao X (2006). Development of human monoclonal antibodies against diseases caused by emerging and biodefense-related viruses.. Expert Rev Anti Infect Ther.

[74] Keller MA, Stiehm ER (2000). Passive immunity in prevention and treatment of infectious diseases.. Clin Microbiol Rev.

[75] Baric RS, Yount B, Lindesmith L, Harrington PR, Greene SR (2002). Expression and self-assembly of norwalk virus capsid protein from venezuelan equine encephalitis virus replicons.. J Virol.

[76] Traggiai E, Becker S, Subbarao K, Kolesnikova L, Uematsu Y (2004). An efficient method to make human monoclonal antibodies from memory B cells: potent neutralization of SARS coronavirus.. Nature Med.

[77] Tian P, Yang D, Jiang X, Zhong W, Cannon JL (2010). Specificity and kinetics of norovirus binding to magnetic bead-conjugated histo-blood group antigens.. J Appl Microbiol.

[78] Chenna R, Sugawara H, Koike T, Lopez R, Gibson TJ (2003). Multiple sequence alignment with the Clustal series of programs.. Nucleic Acids Res.

