# Supplementary material for: Immunogenetic Mechanisms Driving Norovirus GII.4 Antigenic Variation
Source: PLoS Pathog. 2012 May 17;8(5):e1002705. doi: 10.1371/journal.ppat.1002705 (PMC3355092; doi:10.1371/journal.ppat.1002705)
Supplement: Figure S1 — NoV VLP EC50 (reciprocal dilution) in individual plasma samples by VLP. EIA reactivity to individual VLPs is color coded: White; EC50 dilution <100, Purple; EC50 dilution between 100–500, Blue; EC50 dilution between 501–1999, Pink; EC50 dilution ≥2000. Monoclonal antibodies were developed from Donor 302898, named NVB. (PDF) [file ppat.1002705.s001.pdf]

VLP	GI.1.1968/ GI.2.1999	GI.3.1999	GII.3.1999	GII.4.1997	GII.4.2002	GII.4.2006
**Donor Plasma 302898**	140.3	594.8	398.2	2430	155.1	5574
100570	472.3	1222	688.8	995.8	820.4	256.3
100844	498.1	634	656.8	552	335.8	192.9
104697	1163	2877	3666	264.6	121.3	16.41
108412	182	617.8	516.5	400.7	333.2	214.4
104805	309.5	498.8	403.6	786	940.9	280.2
100406	187.5	519.7	656.7	274.6	74.94	119.2
100681	563.6	192.3	133	111.2	70.51	18.38
104041	294.3	840.8	579.9	53.74	60.32	42
96162	31.16	248.6	78.5	697.2	90.47	1675
108546	250.8	186.6	170	721.3	407.6	116.9
100903	250.3	375.1	382.1	363.1	387.4	184.2
104218	203.8	223.9	340.6	353	365.6	139.3
302452	147.6	142.9	150	120.7	140.3	107.4
100486	222	311.2	300.3	306.5	298.6	102.3
108370	158.9	162.2	383.1	141.1	195	69.95
100572	135	416.2	397.1	163.1	148.5	66.36
105123	266.2	256.7	137.4	379.5	230.8	35.57
101757	113.3	157.9	101.3	105.8	50	47.22
104277	83.28	139.3	124.5	270.6	221.1	99.38
101148	94.17	183.3	177	135.2	137.3	84.49
101155	117.5	310.8	272.9	99.22	92.08	49.76
104088	161	156.8	136.9	74.01	85.45	48.33
100630	203.4	227.9	178.9	88.99	80.17	51.3
104359	146.8	189.6	100.7	52.69	49.32	17.49
100233	261.5	228.9	154.5	43.56	neg	16.64
302450	280.5	284.5	265.4	35.41	neg	12.19
102852	84.09	139.7	82.74	121.5	164.7	49.92
105122	67.97	77.29	102.6	271.7	193.8	46.4
104781	91.15	73.74	404	239.1	294.4	50.09
104864	30.18	77.34	44.36	295.3	323.4	233
100210	145.7	132.7	82.59	86.61	neg	92.48

104891	103	100.7	40.89	89.36	80.47	42.26
303215	114.9	179.1	23.7	26.31	neg	27.14
108287	286.6	102.7	77.66	25.9	34.99	20.37
101175	120.4	108.6	95.89	18.85	11.17	12.58
100370	101.6	78.75	129.5	51.32	neg	neg
104900	89.72	467.7	806.4	25.31	25.96	14.96
104475	86.91	273.6	180	56.62	59.04	43.25
104609	93.04	276.9	144.1	52.07	56.26	30.75
100542	33.29	114.6	366.3	64.04	30.59	17.05
100712	61.69	102.6	61.29	106.2	46.54	28.43
305475	43.52	45.79	50.3	259.4	174.7	46.71
303279	57.14	20.8	neg	310	12.96	166.6
104584	143.1	45.39	11.77	62.32	62.02	24.65
100638	169.1	88.11	41.23	56.94	28.59	23.79
100686	129.3	67.26	54.8	44.89	42.35	43.9
104790	54.86	103	60.27	30.47	31.31	27.31
104300	81.07	120.4	59.15	33.6	34.64	21.01
101245	20.76	38.26	142.4	19.11	23.82	14.87
104122	67.79	71.72	94.54	91.71	175.1	76.1
105235	19.34	60.28	31.25	76.53	101.7	28.59
108491	16.55	22.59	14.5	47.32	28.47	12.11
101176	21.13	51.91	34.11	48.92	47.11	18.09
105238	34.45	58.44	40.23	25.95	30.09	17.95
104194	12.83	30.74	22.67	41.08	20.64	neg
105168	18.37	47.22	81.66	19.24	14.16	neg
100117	34.27	15.18	84.72	30.15	neg	neg
100713	neg	23.16	neg	70.09	11.65	neg
104040	neg	13.58	neg	31.84	20.31	neg
100450	neg	neg	neg	25.92	neg	30.01
303105	neg	neg	67.09	neg	neg	neg
105005	neg	neg	neg	neg	neg	neg
