# Supplementary figures and images for: Immunogenetic Mechanisms Driving Norovirus GII.4 Antigenic Variation
Source: PLoS Pathog. 2012 May 17;8(5):e1002705. doi: 10.1371/journal.ppat.1002705 (PMC3355092; doi:10.1371/journal.ppat.1002705)

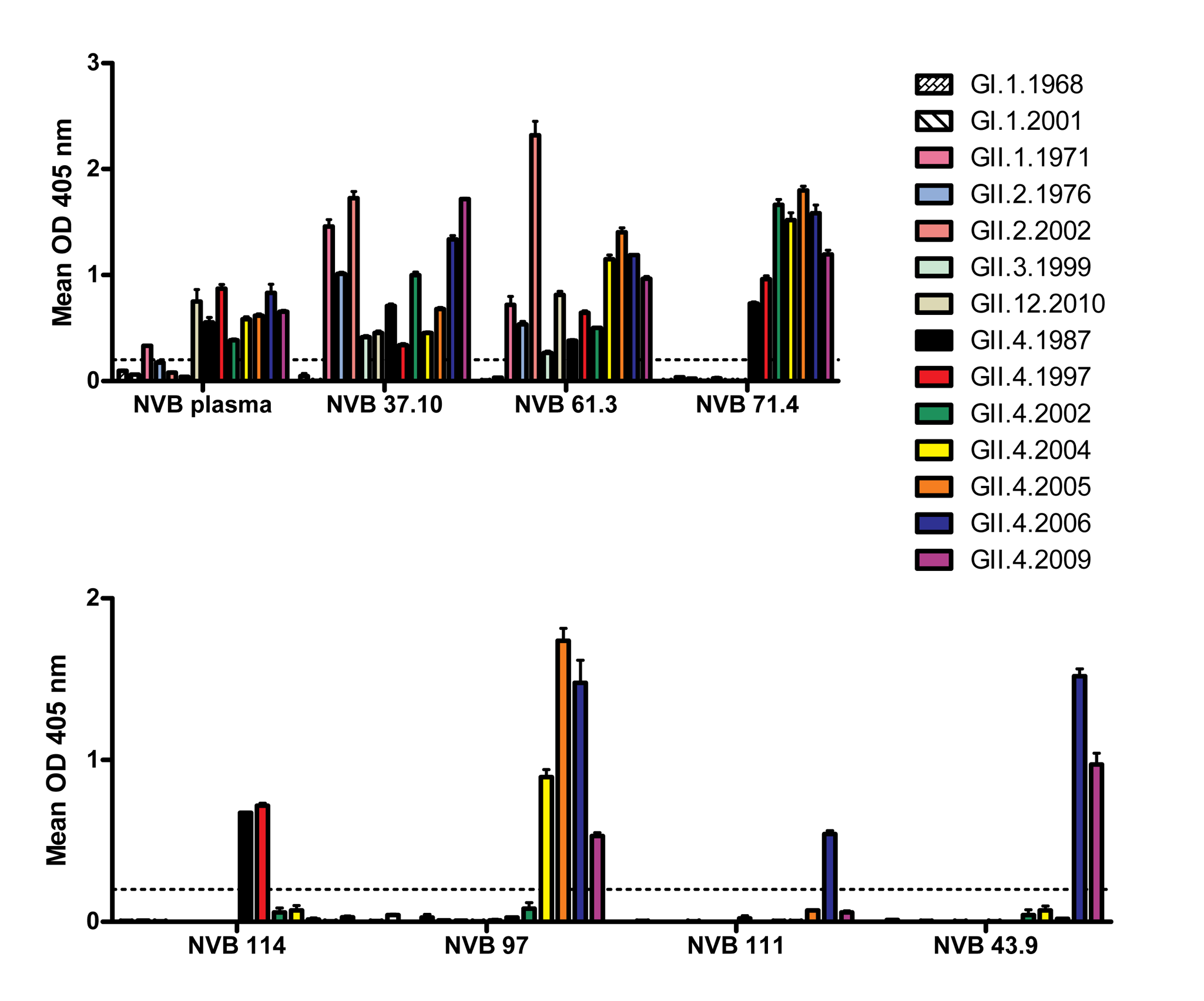

Supplement: Figure S2 — EIA Reactivity of NVB plasma and mAbs against NoV VLPs. Columns present the mean OD of 0.2% NVB plasma or 1 µg/ml mAb reactivity with immobilized VLP. Panel A: Antibodies with broad genogroup II VLP reactivity. Panel B. Antibodies with restricted GII.4 VLP reactivity. Bars are SEM. Mean ODs above 3-fold background (dashed line) were scored as positive. (TIF) [file ppat.1002705.s002.tif]
