# Supplementary material for: Immunogenetic Mechanisms Driving Norovirus GII.4 Antigenic Variation
Source: PLoS Pathog. 2012 May 17;8(5):e1002705. doi: 10.1371/journal.ppat.1002705 (PMC3355092; doi:10.1371/journal.ppat.1002705)
Supplement: Table S1 — Antibody EC50 µg/ml (95% CI) blockade of VLP binding to PGM. (DOC) [file ppat.1002705.s003.doc]

Table S1. Antibody EC50 μg/ml (95% CI) blockade of VLP binding to PGM.

| **Antibody** | **NBV** | **114** | **97** | **111** | **43.9** | **37.10** | **61.3** | **71.4** |
| --- | --- | --- | --- | --- | --- | --- | --- | --- |
| **VLP** |  |  |  |  |  |  |  |  |
| **GII.4.1987** | 0.0673  (0.0634-0.0714) | 0.3414  (0.3024-0.3854) | NB | NB | NB | NB | NB | 0.4506  (0.3914-0.5186) |
| **GII.4.1997** | 0.1506  (0.0973-0.1144) | 0.4152  (0.3647-0.4726) | NB | NB | NB | NB | NB | 13.73  (8.823-21.37) |
| **GII.4.2002** | 0.1786  (0.1572-0.2028) | NB | NB | NB | NB | NB | NB | 1.095  (0.9882-1.212) |
| **GII.4.2005** | 0.1791  (0.1263-0.2538) | NB | 0.1559  (0.1406-0.1729) | NB | NB | NB | NB | 3.544  (2.854-4.399) |
| **GII.4.2006** | 0.0353  (0.0328-0.0381) | NB | 0.1195  (0.1022-0.1396) | 0.7376  (0.6431-0.8459) | 0.1031  (0.0860-0.1236) | NB | NB | 0.9233  (0.7597-1.122) |
| **GII.4.2009** | 0.1102  (0.0952-0.1276) | NB | 0.1810  (0.1728-0.1896) | 9.953  (9.5550-10.37) | 0.1739  (0.1579-0.1915) | NB | NB | 0.2716  (0.2399-0.3075) |

NB; No blockade at 2μg/ml mAb
