# Supplementary material for: Immunogenetic Mechanisms Driving Norovirus GII.4 Antigenic Variation
Source: PLoS Pathog. 2012 May 17;8(5):e1002705. doi: 10.1371/journal.ppat.1002705 (PMC3355092; doi:10.1371/journal.ppat.1002705)
Supplement: Table S2 — Antibody EC50 µg/ml (95% CI) blockade of VLP binding to synthetic biotinylated HBGAs. (DOCX) [file ppat.1002705.s004.docx]

Table S2. Antibody EC50 μg/ml (95% CI) blockade of VLP binding to synthetic biotinylated HBGAs.

| Antibody |  | 114 |  | 97 | 111 | 43.9 | 37.10 | 61.3 |  | 71.4 |
| --- | --- | --- | --- | --- | --- | --- | --- | --- | --- | --- |
| VLP |  |  |  |  |  |  |  |  |  |  |
| GII.4.1987 |  | 0.1054  (0.0952-0.1167) |  | NB | NB | NB | NB | NB |  | 0.0906  (0.0778-0.1055) |
| GII.4.1997 |  | 0.3275  (0.2878-0.3727) |  | NB | NB | NB | NB | NB |  | 0.4338  (0.3983-0.4725) |
| GII.4.2002 |  | NB |  | NB | NB | NB | NB | NB |  | 0.1679  (0.1595-0.1767) |
| GII.4.2005 |  | NB |  | 0.1835  (0.1720-0.1957) | NB | NB | NB | NB |  | 1.219  (1.030-1.443) |
| GII.4.2006 |  | NB |  | 0.0668  (0.0634-0.0703) | 0.3324  (0.2890-0.3823) | 0.05406  (0.0530-0.0552) | NB | NB |  | 0.2039  (0.1909-0.2179) |
| GII.4.2009 |  | NB |  | 0.1732  (0.1608-0.1865) | 2.727  (2.501-2.973) | 0.1140  (0.1003-0.1295) | 0.9753  (0.7247-1.312) | 1.581  (1.229-2.033) |  | 0.3804  (0.3504-0.4130) |

NB; No blockade at 2μg/ml mAb
