# Supplementary material for: Immunogenetic Mechanisms Driving Norovirus GII.4 Antigenic Variation
Source: PLoS Pathog. 2012 May 17;8(5):e1002705. doi: 10.1371/journal.ppat.1002705 (PMC3355092; doi:10.1371/journal.ppat.1002705)
Supplement: Table S3 — NVB plasma (%) and monoclonal antibody (µg/ml) HAI titer. (DOC) [file ppat.1002705.s005.doc]

Table S3. NVB plasma (%) and monoclonal antibody (μg/ml) HAI titer.

| **Antibody** | **Serum** | **114** | **97** | **111** | **43.9** | **71.4** | **37.10** | **61.3** |
| --- | --- | --- | --- | --- | --- | --- | --- | --- |
| **VLP** |  |  |  |  |  |  |  |  |
| **GI.1.1968** | **-** | - | - | - | - | **-** | **-** | **-** |
| **GII.4.2002** | 0.01 | - | - | - | - | - | - | 0.25 |
| **GII.4.2005** | 0.01 | - | 0.13 | - | - | 0.5 | 0.25 | - |
| **GII.4.2006** | 0.001 | - | 0.07 | 0.5 | 0.04 | 0.13 | 0.25 | - |

-; no HAI at tested concentrations
