# Supplementary material for: Immunogenetic Mechanisms Driving Norovirus GII.4 Antigenic Variation
Source: PLoS Pathog. 2012 May 17;8(5):e1002705. doi: 10.1371/journal.ppat.1002705 (PMC3355092; doi:10.1371/journal.ppat.1002705)
Supplement: Table S4 — Plasma (%) and Antibody (µg/ml) EC50 (95% CI) blockade of epitope exchanged VLP binding to PGM. (DOC) [file ppat.1002705.s006.doc]

Table S4. Plasma (%) and Antibody (μg/ml) EC50 (95% CI) blockade of epitope exchanged VLP binding to PGM.

| **Antibody** | **NBV** | **97** | **114** | **111** | **43.9** |
| --- | --- | --- | --- | --- | --- |
| **VLP** |  |  |  |  |  |
| **GII.4.1987** | 0.0673  (0.0634-0.0714) | NB | 0.3414  (0.3024-0.3854) | NB | NB |
| **GII.4.1987/2006A** | 0.0167  (0.0184-0.0152) | - | NB | 1.152  (1.343-0.9889) | 0.0366  (0.0304-0.0437) |
| **GII.4.1987/2006B** | 0.0457  (0.0398-0.0525) | - | 0.1970  (0.1746-0.2222) | - | - |
| **GII.4.1987/2006C** | 0.0402  (0.0348-0.0464) | - | 0.2209  (0.1882-0.2594) | - | - |
| **GII.4.1987/2006D** | 0.0599  (0.0550-0.0652) | 0.6349  (0.5530-0.7291) | 0.4948  (0.4249-0.5762) | - | - |
| **GII.4.1987/2006E** | 0.2025  (0.18070.2269) | - | 0.5587  (0.4757-0.6560) | - | - |
| **GII.4.2006** | 0.0353  (0.0328-0.0381) | 0.1195  (0.1022-0.1396) | NB | 0.7376  (0.6431-0.8459) | 0.1031  (0.0860-0.1236) |
| **GII.4.2006/1987A** | 0.2770  (0.2418-0.3173) | - | NB | NB | NB |
| **GII.4.2006/1987B** | 0.0522  (0.0435-0.0627) | - | - | - | - |
| **GII.4.2006/1987C** | 0.0416  (0.0362-0.0480) | - | - | - | - |
| **GII.4.2006/1987D** | 0.0383  (0.0364-0.0403) | NB | - | - | - |
| **GII.4.2006/1987E** | 0.0991  (0.0823-0.1194) | - | - | - | - |

NB; No blockade at 2μg/ml mAb

-; not tested
